# Intraspecific variation in growth response to drought stress across geographic locations and genetic groups in *Coffea canephora*

**DOI:** 10.1002/ece3.9715

**Published:** 2023-01-03

**Authors:** Catherine Kiwuka, Jan Vos, Jacob C. Douma, Pascal Musoli, John W. Mulumba, Valérie Poncet, Niels P. R. Anten

**Affiliations:** ^1^ Centre for Crop Systems Analysis Wageningen University Wageningen The Netherlands; ^2^ Plant Genetic Resources Centre National Agricultural Research Organization Entebbe Uganda; ^3^ National Coffee Research Institute National Agricultural Research Organization Mukono Uganda; ^4^ UMR DIADE, IRD Univ. Montpellier Montpellier France

**Keywords:** drought stress, growth tolerance trade‐off, intraspecific variation, local adaptation *Coffea canephora*

## Abstract

Uganda lies within the drier end of the natural distribution range of *Coffea canephora* and contains unexplored genetic material that could be drought‐adapted and useful for developing climate‐resilient varieties. Using water treatment: (i) ample and (ii) restricted‐water, the response of 148 genotypes were studied comprising wild, feral and cultivated *C. canephora*. Biomass allocation, standing leaf area and leaf area growth data were collected. Linear mixed effect models and PCA were used to the analyze effect of water treatment on genotypes from different: (i) cultivation status, (ii) genetic groups and (iii) locations. We also assessed the relationship between drought tolerance for relative growth rate in leaf area (RGRA), total number of leaves (TNL), total leaf area (TLA) and total leaf dry weight (TLDW) of genotypes at final harvest. Restricted‐water reduced RGRA across genetic groups (3.2–32.5%) and locations (7.1–36.7%) but not cultivation status. For TNL, TLA and TLDW, genotypes that performed well in ample‐water performed worse under restricted‐water, indicating growth‐tolerance trade‐off. Drought tolerance in RGRA and TNL were negatively correlated with wetness index suggesting some degree of adaptation to local climate. Findings indicate a growth‐tolerance trade‐off within this tropical tree species and drought tolerance of Uganda's *C. canephora* is somewhat associated with local climate.

## Graphical abstract

Drought tolerance within *C. canephora* is negatively linked to growth. Drought tolerance of Uganda's *C. canephora* is weakly though significantly linked to the local climate. Intraspecific diversity, associated with adaptation to local conditions, can be exploited in breeding for climate‐resilient cultivars.

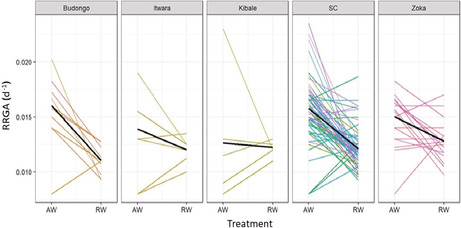

## 1 INTRODUCTION

Water availability is a major factor limiting global coffee production largely because of the drought sensitivity of *Coffea* species and because a large fraction of the production is sustained by small‐holder farmers who usually lack resources to establish irrigation facilities (Craparo et al., 2015; DaMatta & Cochicho Ramalho, 2006; Wintgens, 2009). Problems of water limitation in coffee production are expected to be aggravated by climate change. This is because, across the coffee production belt, a temperature increase of 2.1°C has been predicted by 2050 (IPCC, 2014; Parry et al., 2007) and this warming can directly result in increased vapor pressure deficits, higher potential evapotranspiration and hence drought stress in plants. Indirectly, the increase in global average temperatures is expected to result in shifts in the annual precipitation with more frequent occurrences of severe droughts (Schiermeier, 2008). The changes in temperature and precipitation together may have strong negative effects on coffee production (Bunn et al., 2015), although Verhage et al. (2017) reported that the CO_2_ fertilization effect arising from elevated CO_2_ concentrations could offset the negative effects of climate change in average coffee yields by a small net increase. The global distribution and production of coffee are therefore likely to be significantly affected by climate change (DaMatta & Cochicho Ramalho, 2006; Davis et al., 2012; Jassogne et al., 2013). There is a need for finding or developing drought‐tolerant genotypes, and one way of working toward this is to explore the natural diversity in wild coffee populations.

*Coffea canephora* Pierre ex A. Froehner is a tree native to African tropical lowland forests stretching from Guinea in West Africa through the Congo River Basin to Uganda in East Africa (Berthaud, 1986; Coste, 1992; Davis et al., 2006; Montagnon et al., 1992). Generally, these tropical forests are characterized by abundant rainfall (precipitation >2000 mm year^−1^) with a short or no dry season, high atmospheric humidity and stable average temperatures between 24°C and 26°C (Coste, 1992; DaMatta, 2018; DaMatta & Cochicho Ramalho, 2006). However, even in these moist tropical forests, there occur periodic water shortages due to dry spells (Engelbrecht et al., 2006). Furthermore, the natural geographical distribution of *C. canephora* extends into the somewhat drier areas (Masih et al., 2014), e.g., in Uganda. Tree growth (e.g., biomass or leaf area increment, referred to as performance hereafter) is commonly observed to decrease with drought intensity (Chapin, 1980; Garnier & Poorter, 2007; Grime & Hunt, 1975). Across tree species (at the interspecific level), there tends to be a negative correlation between growth under well‐watered conditions and drought tolerance which is defined as the extent to which plants can maintain these growth rates under water‐stressed conditions (i.e., drought tolerance in growth, the ratio of growth under stressed and unstressed conditions) (Chapin, 1980; Garnier & Poorter, 2007; Ouédraogo et al., 2013). Growth and survival under dry conditions tend to be associated with traits such as low specific leaf area (leaf area/mass ratio), fewer or smaller stomates, small stem vessel diameter, high fractions of dry mass in roots, low leaf area to root mass ratio and low leaf area to sapwood ratio which tend to reduce growth rates under well‐watered conditions (Lambers et al., 2008).

While multispecies comparisons are useful to understand ecological strategies and community composition, questions regarding natural selection and applications for breeding require additional intraspecific comparisons across wild accessions of a species. When an environmental stress gradient such as water availability acts as a selective force, one may expect tolerance of a genotype to this stress factor to be related to the climate in the site of origin (Alberto et al., 2013). Analyzing such patterns is important as it may provide insights into natural selection but may also provide basic information to assess the adaptive potential to climate change and, for crops, identify drought‐tolerant genotypes (Alberto et al., 2013; Rungwattana et al., 2018). However, very few studies have compared wild accessions from different climates for tropical trees such as coffee. Rungwattana et al. (2018) compared wild accessions of rubber (*Hevea brasiliensis*) from different locations across a rainfall gradient in the Amazon Forest and found no correlation between any of the traits investigated and either temperature or rainfall at the site of origin. In *C. canephora'*s congener, *C. arabica*, comparisons between nine accessions from different Ethiopian forests showed that accessions from drier areas were more plastic in leaf gas exchange traits in response to changes in water availability than those from wetter areas (Beining, 2007) but another study with a similar set of accessions found no correlations between water availability as an experimental factor and leaf gas exchange traits (Kufa & Burkhardt, 2011).

Uganda has been reported to have substantial *C*. *canephora* diversity (Kiwuka, 2020; Kiwuka et al., 2021; Musoli et al., 2009; Ngugi & Aluka, 2019) which could be explored to identify functional diversity in regard to drought stress. But to our knowledge, intraspecific comparisons of drought‐related traits in *C*. *canephora* have been limited to cultivated material (DaMatta et al., 2003; Dias et al., 2007; King'oro, 2014; Menezes‐Silva et al., 2015; Pinheiro & Var, 2004; Silva et al., 2013). While the aforementioned studies give important insights into the morphological and physiological drivers of drought tolerance, exploration of the variation in drought tolerance across wild populations and potential correlations with climate need to be done. Furthermore, none of the studies on tropical trees has explored the extent to which drought tolerance is associated with genetic diversity, a link that would provide helpful information to interpret drought adaptation. Finally, as far as we know, drought tolerance in coffee has also not been explored along a cultivation status trajectory, i.e., comparing wild, feral (second generation or higher of formerly cultivated material and abandoned for over 50 years) and cultivated genotypes. It is therefore unknown whether the cultivation of *C. canephora* has been selected for or against drought tolerance.

This study was set out to determine: (i) the effect of water treatment on vegetative growth (biomass and leaf area increment) of *C. canephora* genotypes, collected across a climatic gradient in Uganda and categorized by (a) cultivation status, (b) genetic groups as characterized by Kiwuka et al. (2021) where Uganda's native *C. canephora* is categorized into four distinct genetic clusters comprising genotypes from Zoka, Budongo, Itwara and Kibale forests of northwestern (NW) zone, and one large genetic cluster including genotypes from Malabigambo, Mabira, and Kalangala forests of the southern‐central (SC) as well as the feral and cultivated accessions, (c) and location, indicating the different climatic envelopes (as defined by location specific bioclimatic variables for the years 1950–2000), (ii) the relationship between performance under restricted and ample‐water conditions, (iii) the relationship between drought tolerance of genotypes and wetness index (WI) at their native location. WI, the ratio of mean annual precipitation to mean annual potential evapotranspiration (PET) is a reasonable proxy for local climate wetness, whereby high WI indicates wetter climates and vice versa (note that we do not use the original but confusing term, aridity index, from Zomer et al. (2008)). We hypothesized that, since Uganda's wild *C. canephora* populations occur in different climatic envelopes, genotypes from dry (lower WI) locations characterized by high temperatures, low precipitation, and high PET and will have comparatively higher growth and performance under restricted‐water conditions than genotypes from locations with low to moderate temperatures, high precipitation, higher WI and low PET (wet location). Additionally, we expect a trade‐off between drought tolerance and performance, whereby the mechanisms that underlie drought tolerance in material from dry locations are associated with slow growth and the inability to exploit favorable conditions (Amissah et al., 2018; Lambers et al., 2008; McGill et al., 2006; Sade et al., 2012).

## 2 MATERIALS AND METHODS

### 2.1 Plant material

A total of 228 genotypes of *Coffea canephora* Pierre ex A. Froehner were collected from the wild and the National coffee germplasm collection fields in 2014 (Kiwuka et al., 2021). Each genotype was categorized according to three main sets of determinants (factors): (1) cultivation status, (2) genetic group and (3) location.

Cultivation status was defined based on the level of management of the material and included three levels: (i) wild‐plant material collected from tropical natural forests and free from direct human management, (ii) feral‐ material collected from formerly cultivated and currently abandoned (abandoned for at least 50 years) coffee fields. Caution was taken not to collect from trees that were older than 15 years, as a way of ensuring that feral materials are sampled from trees that were belonging to at least the second generation of the abandoned coffee fields and (iii) cultivated; a subset represented by material collected from assembled *C. canephora* germplasm fields at the National Agricultural Research Organization (NARO) institutes located at Kawanda and Kituza. The sampled cultivated material represented the range of traditional and commercial *C. canephora* diversity in Uganda's Robusta coffee cultivation and breeding system.

The second main category was genetic groups. Ugandan *C. canephora* diversity (Genetic group (O)) has been reported to be distinct from other known genetic groups at the species level (Kiwuka et al., 2021; Merot‐L'anthoene et al., 2019; Musoli et al., 2009). Ugandan *C. canephora* diversity uniquely differentiates into two main subgroups namely: (i) The Southern Central (SC), (ii) the North Western (NW) groups; the latter of which further differentiates into four groups corresponding to four forest locations (Itwara, Kibale, Budongo and Zoka) (see Table S1) (Kiwuka et al., 2021). The third category was geographic location. Uganda is categorized into 16 homogeneous climatological zones based on precipitation patterns (Basalirwa, 1995) and the country's *C. canephora* diversity occurs in five of these 16 distinct climatic zones (see Table 1; Figures S1 and S2). The study materials were collected from nine locations in the five distinct climatic zones (Table 1). Each location was defined based on its geographical position and administrative boundaries: (i) Budongo; (ii) Itwara; (iii) Kalangala; (iv) Kibale; (v) Mabira; (vi) Malabigambo and (vii) Zoka (Table 1; Figures S1 and S2). Genotypes from Kituza and Kawanda were not included in this category because plants grown there were collected from other places. Regarding the environmental gradient across locations, NEMA (2009) showed that Zoka is at the driest and Kalangala at the wettest end of the range.

**TABLE 1:** Description of collection location of *Coffea canephora* study material

Location (code)	Geo‐reference	Cultivation status	No. of genotypes	Climatic zones	PET (mm year^−1^)	WI	Annua l mean temper ature (°C)	Annua l precipi tation (mm)
Budongo (BD)	01°43′27″ N 31°32′45″ E	Wild	16	K	1740	0.76	23	1322
Itwara (IT)	00°47′29″ N 30°28′19″ E	Wild	10	L	1604	0.89	20	1422
Kalangala (KL)	00°26′ S 32°15′ E	Wild & feral	19	A1	1560	1.25	21	1942
Kawanda (KW)	0°24′30.42″ N 32°32′09″ E	Cultivated	19	B	1624	0.76	22	1238
Kibale (KB)	00°30′ N 30°24′ E	Wild	9	L	1637	0.77	20	1267
Kituza (KT)	0°15′26.81″ N 32°47′27.7″ E	Cultivated	28	B	1573	0.93	21	1464
Mabira (MB)	0°23′54″ N 33°0′59″ E	Wild	15	B	1652	0.82	22	1356
Malabigambo (ML)	00°57′7′′ S 31°38′25′′ E	Wild	7	A1	1604	0.88	21	1414
Zoka (ZK)	03°01′03.0″ N 31°39′21.0″ E	Wild	25	J	1869	0.68	24	1267

*Note*: Climatic zones as classified by Basalirwa (1995) A1, B, K, L, J (see Figure S1); PET values were estimated following Hargreaves and Samani (1985) who use mean monthly temperature, mean monthly temperature range and mean monthly extra‐terrestrial radiation (Zomer et al., 2008) while WI (wetness index); (Aridity index in Zomer et al. (2008)) defined as a ratio of mean annual precipitation to mean annual potential evapo‐transpiration (PET). Note that genotypes from Kawanda and Kituza are not in the analysis of location effects.

### 2.2 Sampling strategy

A hierarchical sampling strategy was employed to collect samples (stem cuttings) from the different locations. Wild genotypes were sampled from seven tropical natural forests: (i) Budongo forest, (ii) Itwara Central Forest Reserve, (iii) Kalangala (Lutoboka central forest reserve), (iv) Kibale forest national park, (v) Mabira forest reserve, (vi) Malabigambo forest and (vii) Zoka forest. In each location (forest) except Kalangala, samples were collected from five sub‐sites that were separated by distances of at least 5 km. From each sub‐site, five healthy *C. canephora* trees were identified from which we collected stem cuttings. Since *C. canephora* is an allogamous species, each sampled plant was considered to be genetically unique and therefore, each sampled tree was regarded as a distinct genotype in this study. The assumption that each sampled tree is a unique genotype was confirmed by genetic analysis by Kiwuka et al. (2021). Contrary to other locations, the Kalangala site comprised remnants of natural forest systems and secondary forests regenerated from formerly cultivated coffee fields, and therefore, the coffee populations in Kalangala were considered wild or feral depending on where they were collected from. Samples that were collected from natural forest fragments were regarded as wild while samples from collected abandoned cultivation fields were considered feral.

The cultivated samples were collected from two germplasm field collections of the Ugandan National Agricultural Research Organization (NARO): National Coffee Research Institute; Kituza and the National Agricultural Research Laboratories at Kawanda. The cultivated genotypes were selected based on their historical and passport data to represent the total range of traditional and commercially cultivated *C. canephora* diversity, including the two predominant forms found in Uganda: Erecta, or upright forms, and Nganda, or spreading forms (Thomas, 1935) and the six elite clones, namely: KW13, KW14, KW15, KW16, KW18 and KW19 (details can be found in Kiwuka et al. (2021)).

### 2.3 Stem cutting establishment

All the collected stem cuttings were rooted in a screen house at the National Agricultural Research Laboratories (NARL), Kawanda at 0°25′ N, 32°32′ E, 1195 m a.s.l., starting on 30th May 2015. The establishment of the material from stem cutting followed a tested protocol by the National Coffee Research Institute (NaCORI, unpublished). The collected stem cuttings were cut into 7 cm inter‐nodal wood cuttings with one pair of leaves. A total of 7419 inter‐nodal cuttings, for all the collected genotypes (230) were planted in polypots and placed in transparent plastic cages for root establishment. The number of cuttings per genotype ranged from 7–99 the median being 33. The polypots had a diameter of 5 cm and a height of 7 cm and were filled with a mixture of topsoil, sand and manure in a ratio of 3:2:2 by volume. Before planting, each stem cutting was dipped in rooting hormone (Seradix ‘2’, 0.8% w.w, IBA; Twiga Chemicals Industries, Nairobi, Kenya) to boost their rooting potential. After 7 months, the young plants that had grown from the cuttings were hardened off and, transferred into 10 L pots. The potting medium comprised of black loamy forest soil, lake sand and decomposed cattle manure in the ratio of 3:1:1, with a volumetric water content of 30% (±0.22) at field capacity and 6% (±0.16) at permanent wilting point respectively (See details of the chemical and physical properties of the potting medium in Data S1). Ten grams of an inorganic compound fertilizer comprising: 25% nitrogen, 5% phosphorous, 5% potassium and 5% of sulfur of the total weight of the elements in the fertilizer was added per pot. Pots were optimally irrigated for 6 months before starting the experimental treatments.

### 2.4 Experimental design

Out of the 230 collected genotypes, 148 produced a sufficient number (≥5) of properly rooted plantlets to start the experiment with. From October 10th to 15th 2016, 16 months after re‐planting the stem cuttings, 1184 rooted plants were arranged into a split‐plot design; with two watering regimes (ample vs restricted‐water) as the main factor and the different *C. canephora* genotypes as the sub‐factors. Plants were grown in a ‘rain out’ screen house (40 m by 6.5 m) that was blocked into four sections, based on the variation in radiation that was visually assessed (148 remaining genotypes × four blocks (with each split into two) × two water regimes (ample and restricted)).

To establish ample vs restricted‐water availability treatments, we assessed the potting medium's properties, e.g., water content at field capacity, permanent wilting point and the daily evapotranspiration rates within the screen house by weighing over time a selection of 10 pots. Soil water loss was also estimated by monitoring soil moisture content in pots using a soil moisture sensor (Trime‐Pico 64/32, HD2 IMKO Micromodultechnik, Ettlingen, Germany). The ample‐water treatment was set at 25 v% which was about 80% of soil moisture content at field capacity, while the drought‐stressed regime (restricted‐water hereafter) was sustained at 10 v% soil moisture at the permanent wilting point.

Plants in the ample‐water treatment received on average 1000 ml of water per watering interval, which was, on average, once a week. Plants in the restricted‐water treatment were subjected to gradually increasing severity of drought stress and the basic regime was that on average, plants received 300 ml per week for the first month, 300 ml per fortnight for the following month, a one‐time 300 ml water gift in the third month and finally a month without water. To minimize the potential plant‐size drought bias, i.e., the fact that larger plants consume more water and are therefore exposed on average to drier conditions, the following procedure was used: in the initial experimental phase, a sub‐set of plants (54 plants; selected to represent the architectural [number of leaves, number of primary branches, number of suckers and leaf area], variation across the experiment) were monitored to determine their soil water content (both gravimetrically and with the soil moisture probe) every week and their corresponding number of leaves, number of primary branches, the number of suckers and leaf area were non‐destructively estimated. The leaf area of fully expanded leaves was estimated from leaf length and width using the linear model (area per leaf = leaf length × leaf width × *k* (*k* = correction factor = 0.66)) of Schmildt et al. (2015). These data yielded a correlation between leaf area and water loss and the relation was used as a guide to determine the frequency of watering for every plant based on its leaf area. This procedure ensured that size‐dependent effects on the actual soil moisture experienced by plants were minimized. At the end of the experiment, it appeared that the amount of water supplied (W (ml)) could be linearly related to the leaf area (LA (cm^2^)) to each plant was described by the formula: *W* = 1479 + 0.178 LA, *p* = .000 and *R* ^2^ = 0.27.

The experimental treatment period lasted 4 months (from plant age 20 months to 24 months; age zero is when the stem cuttings were planted to root). Data on temperature and relative humidity in the screen‐house were recorded by sensors with data logging (Tinytag logger Plus 2 Dual Channel Temperature/Relative Humidity, TGP‐4500; Gemini data loggers Ltd., Chichester, Chichester West Sussex, UK) on an hourly basis. The average daily temperatures and relative humidity of the screen‐house throughout the experimental treatment period were: 23.1°C (±4.3) and 83.1% (±18.0) respectively while average daily vapor pressure deficit estimates were 0.49 (±0.15).

### 2.5 Data collection

Data were collected at three stages: (i) at the start of the treatment phase; (ii) during the treatment phase and (iii) at the end of the treatment phase (Table S2). At the start of the treatment phase on 25th May 2017 (plant age 20 months) several non‐destructive measurements were done to provide a baseline for later size increment measurements: plant height, number of nodes, number of leaves (fully grown and proportion/fractions from estimated full size of developing ones), length and width of fully expanded leaves and stem diameter at 5 cm from the base. After these measurements, the youngest fully expanded leaf pair was marked, to establish a recognition point for measuring new growth. The second data collection stage (at the point when 10% of the plants subjected to restricted‐water started to exhibit leaf wilting (scored visually)), was taken from 21–24 June 2017. The final measurement occasion, at the end of the treatment phase, was conducted on 12–26 September 2017, with measured traits as listed in Table S2.

### 2.6 Methods to measure plant properties

Plant height was measured using a meter ruler from the base (point of origin from the cutting) to the last node. To estimate the area per leaf and subsequently the total leaf area, we used the same model as that used for determining leaf area about the watering regimes, i.e., we measured length and width and then used the linear model (leaf length × leaf width × *k* (correction factor)) of (Schmildt et al., 2015) on all fully unfolded leaves and obtained a correction factor (*k* of 0.66) that was used on all measured leaves. The leaf area on the main stem was measured in this way for all plants. But due to the necessity to reduce the workload, the leaf area of primaries and suckers was measured using the aforementioned linear model, but only for all plants in one block. For every genotype, the leaf area of primaries and suckers in blocks two, three and four were estimated from the ratio of leaf fresh weight to leaf area, generated from the measured plants in block 1. At the end of the experiment, for each plant, leaves were separated into leaves from the main stem, primaries and suckers. To obtain total leaf fresh weight (TLFW) and total leaf dry weight (TLDW), the fresh weight of all leaves was estimated by weighing fresh leaves while leaf dry weights were measured after oven drying (70°C to a constant weight).

Specific leaf area (SLA) was estimated as the ratio of leaf area and leaf dry weight accumulated within the experimental treatment phase. The roots of each plant were harvested and cleaned under running water and on a wire mesh. Using the water displacement method, fine roots (excluding the taproot with a diameter larger than 3 mm) were dipped in a measuring cylinder to estimate their root volume. The root volume and total leaf area (TLA) of each plant were used to estimate the root volume to leaf area ratio (RL). Four growth‐related traits were used to characterize the genotype responses to drought stress. These were relative growth rate in leaf area (RGRA, see below for how this was calculated), the total number of leaves (TNL) total leaf area (TL (cm^2^)), total leaf dry weight (TLDW (g)), specific leaf area (SLA (cm^2^ g^−1^)) and root volume to leaf area ratio (RL (cm^3^ cm^−2^)). Note that all traits, except root volume, refer to growth during the experimental period, excluding the biomass at the start of treatments. RGRA was used to assess in more detail the cultivation status, location and genotype response to restricted and ample availability of water. Relative growth rates were used for two reasons: (i) to reduce confounding effects of initial plant size and (ii) we dealt with very young plants for which the assumption of them being in the exponential growth phase was reasonable. We focused on area, dry mass and number of leaves because of practical reasons (measurable non‐destructively; base measurements of biomass were not available) and because leaf area determines light interception capacity, photosynthesis and subsequent growth (Poorter & Remkes, 1990; Weraduwage et al., 2015) (and in coffee fast vegetative growth are typically associated with high yields (Cilas et al., 2006)).

RGRA was calculated as

(1) $$\text{RGRA}=\frac{\text{In}\left({\mathrm{LA}}_{E}-{\mathrm{LA}}_{I}\right)}{t_{E}-t_{I}}$$

where LA_E_ and LA_I_ are leaf area at *t*_E_ – and *t*_I_, respectively.

The difference *t*_E_ – *t*_I_ reflects the 84 days between the start of the treatment phase (*t*_I_) and the day of the final harvest (*t*_E_).

Drought tolerance was defined as the capacity of a genotype to maintain its growth under drought stress (restricted‐water) and was computed per genotype as the ratio of the trait mean in restricted‐water to the trait mean in ample‐water across blocks.

### 2.7 Data analysis

All the analyses and plots done in the study were performed using R version 3.5.0 (R Core Team, 2018). Linear mixed effect models were applied to test the effects of water treatment on selected growth traits across (i) cultivation status, (ii) genetic group or (iii) location. Linear mixed‐effect models were used because mixed models account for unbalanced, nested designs (such as varying numbers of genotypes by cultivation status, genetic groups and location) that occurred in our data (Bates et al., 2015). To estimate the impact of water shortage on plant traits across cultivation status, genetic groups and locations, genotypes were considered a random effect both in terms of the intercept: i.e., the absolute trait value in ample‐water, and the slope: i.e., the response to drought (difference between the trait value in ample and restricted‐water conditions). To account for the heterogeneity of variance in the observations, variances in the traits were dependent on the cultivation status, genetic group or location (Zuur et al., 2010).

The model with cultivation status had 12 parameters: three levels of cultivation status (CS) and two water treatments (making six parameters), three parameters of the random effect to model differences across genotypes: (i) a parameter to model the variation of traits in ample‐water conditions (intercept), (ii) a parameter related to the variation in the treatment effect (slope), and (iii) a parameter that models the correlation between the intercept and the slope, and three parameters to account for a different residual variance per cultivation status (see Model 1 in Data Box S1). The model with the genetic group had 18 parameters: two for each genetic group (making 10) and three parameters of the random effect to model differences across genotypes: (i) a parameter to model the variation of traits in ample‐water conditions (intercept), (ii) a parameter related to the variation in the effect of the treatment effect (slope), and (iii) a parameter that models the correlation between the intercept and the slope, and five parameters to account for a different residual variance per genetic group (see Model 2 in Data S1). Note that while testing the genetic group effect, all genotypes that were misclassified and/or hybrids were not considered.

Including factor location in the analysis enabled us to test for differences in terms of the environment but also for genetic basis, and therefore, indirectly for putative local adaptation. Therefore, the model with location had in total 24 parameters, two for each location (14) and three parameters of the random effect to model differences across genotypes: a parameter to model the variation of traits in control (intercept), a parameter related to the variation in the treatment effect (slope), a parameter that models the correlation between the intercept and the slope, and seven parameters to account for a different residual variance per location (see Model 3 in Data Box S1).

Post‐hoc Tukey tests were performed to determine: (i) groups with statistically significant mean differences in their performance (RGRA) due to the water treatment across cultivation status, genetic groups and location; (ii) groups of cultivation status, genetic groups or location which responded significantly differently to the water treatment and (iii) groups with significant differences in absolute performance under ample‐water and restricted‐water conditions across cultivation status, genetic groups and locations. Tukey adjustment to *p*‐values was done in case of multiple comparisons. The linear mixed model analyses were performed using packages “nlme” (Pinheiro et al., 2017), “emmeans”(Lenth et al., 2018) and the plots were generated using “ggplot2”(Wickham et al., 2016) packages. For all the analyses, any effect with *p* < 0.05 was considered statistically significant and non‐significant at *p* > 0.05.

#### 2.7.1 Multivariate analysis of growth‐related traits

To explore the multivariate dependency between the measured traits, a principal component analysis was performed on the genotypic means. Only genotypes were included for which there were at least two replicates available. All variables were standardized to a mean of zero and scaled to unit variance before the analysis at both treatment levels. Next, to test whether location and cultivation significantly affected the suite of traits, a multivariate analysis was performed using a PERMANOVA. These PERMANOVA tests, similar to a classical multivariate ANOVA, whether the dissimilarities between genotypes from the same location, status and treatment are smaller than the dissimilarities between genotypes across locations, status and treatment (Anderson, 2001). We used Euclidean distances between the centred and scaled observations, and 999 permutations.

#### 2.7.2 Drought tolerance performance trade‐off

Type (II) major axis regression was performed to determine the relationship between the genotypic average growth trait in ample‐water versus restricted‐water. Type II regression was used to account for both measurement errors in the independent and the dependent variable (David & Neville, 2002) and to test whether the slope and intercept were different from each other. The analysis was performed using the package “smatr” (Warton et al., 2012).

#### 2.7.3 Drought tolerance climate relationship

In addition, a weighted linear regression analysis was performed to determine the relationship between drought tolerance based on RGRA_,_ TNL and TLDW and wetness index (WI). The analysis was performed in R version 3.5.0 Statistical Software. Because the number of replicates varied across genotypes in locations, we introduced weights for replicates in the analysis. In this weighted linear regression analysis, we excluded genotypes from Kawanda and Kituza because the genotypes in these collections were sourced from different origins and assembled as ex‐situ collections at NARO institutes, and therefore, we could not retrieve the WI of these genotypes. The probability of rejecting the null hypothesis that there is no relationship between performance in low‐water conditions and wetness index (WI) or no relationship between drought tolerance and wetness index (WI) was set at *p*‐value >.05. The weighted linear regression models were fitted with lm () functions in R version 3.5.0 Statistical Software.

## 3 RESULTS

The study results are presented in hierarchical order starting with: (i) the effect of water treatment on the grand mean of growth response traits (i.e., lumping genotypes together), (ii) the main effects of factors, i.e., cultivation status, genetic groups and location on growth response traits, (iii) the detailed synthesis of the effect of water treatment on RGRA as our proxy trait for plant performance, (iv) the relationship between performance under ample and restricted‐water conditions, (v) the relationship between performance under restricted‐water conditions and wetness index of the locations and (vi) the relationship between drought tolerance and wetness index of the locations.

### 3.1 Main effects of water treatment on growth response traits

The water treatment significantly affected all the studied traits (Tables 2 and 3). Relative growth rate in leaf area (RGRA (d^−1^)), total number of leaves (TNL), total leaf area (TLA (cm^2^)), total leaf dry weight (TLDW (g)) and specific leaf area (SLA (cm^2^ g^−1^)) were on average (12–38%) lower in the restricted‐water than in the ample‐water (Table 2). The larger declines for TNL, TLA than in TLDW in the restricted water treatment is consistent with the negative effect of restricted‐water on SLA. Root volume to leaf area ratio (RL (cm^3^ cm^−2^)) was higher in restricted‐water conditions than in ample‐water conditions (Table 2), indicating a shift in the partitioning of resources toward root growth in restricted‐water conditions.

**TABLE 2:** Effect of water treatment on the mean and standard error of selected growth response traits of *C. canephora*

Trait	Ample‐water grand mean (SE)	Restricted‐water grand mean (SE)	Relative change (%)
RGRA (d^−1^)	0.016 (0.0001)	0.012 (0.0001)	−25.0
TNL	21 (0.5)	13 (0.3)	−38.1
TLA (cm^2^)	3653 (94)	2526 (53)	−30.9
TLDW (g)	17 (0.5)	13 (0.4)	−23.5
SLA (cm^2^ g^−1^)	251 (5)	221 (3)	−12.0
RL (cm^3^ cm^−2^)	0.007 (0.0002)	0.009 (0.0002)	28.6

Abbreviations: RGRA (d^−1^), Relative growth in leaf area; TNL, Total number of leaves; TLA (cm^2^), Total leaf area; TLDW (g), Total leaf dry weight; SLA (cm^2^ g^−1^), Specific Leaf area; RL (cm^3^ cm^−2^), Root volume to leaf area ratio; SE, standard error.

### 3.2 Interaction effects of water treatment with cultivation status, genetic group and location on growth response traits

In the linear mixed model analysis, the effects of factors (i.e., cultivation status, genetic group and location) varied across growth response traits (Table 3). The cultivation status did not have significant effects on RGRA, TNL and TLDW (*p* > .05), but did significantly affect SLA and RL (Table 3). On average, wild genotypes had the highest SLA (244 cm^−2^ g^−1^) but the difference was only significant with the feral and not with the cultivated genotypes (Table S3). For RL, cultivated genotypes had a significantly higher average value (0.0087 cm^3^ cm^−2^) than wild and feral genotypes and there were no significant differences between wild and feral RL values (Table S3). There were no significant interaction effects between cultivation status and treatment for any of the selected traits, except for TLA indicating that only for TLA, the treatment effect differed across cultivation status. Under ample‐water conditions, cultivation status had no significant effects on TLA while under restricted‐water conditions wild genotypes, had a significantly lower TLA than feral and cultivated genotypes whose TLA's were not significantly affected by water availability. In the restricted‐water treatment, wild genotypes had the lowest average TLA which was 24.4% lower than the highest average TLA observed in feral genotypes (Table S3). These findings suggest that in terms of TLA, wild genotypes might be more sensitive to low water availability than non‐wild genotypes.

**TABLE 3:** Significance of effects of the factors on the growth response traits of *C. canephora* number in the table are *F*‐values of linear mixed models testing the effect of factors on performance

Factors	RGRA	TNL	TLA	TLDW	SLA	RL
Cultivation status (CS)						
Treatment	136.367***	60.32***	27.19***	14.48***	15.93***	26.70***
CS	0.91	1.05	1.63	***2.41***	4.75*	3.33*
Treatment*CS	0.74	0.71	3.25*	1.47	0.20	0.80
Genetic group						
Treatment	117.79***	62.61***	35.67***	16.51***	12.03***	14.47***
Genetic group	1.29	2.32	6.37***	8.77***	1.66	7.58***
Treatment*Genetic group	2.76*	***2.02***	1.18	0.93	0.34	0.48
Location						
Treatment	111.17***	63.26***	46.20***	16.68***	8.79*	27.7***
Location	2.39*	2.00	9.31***	10.78***	5.50***	1.05
Treatment*Location	3.20 *	2.93*	3.85***	***2.15***	1.08	***2.03***

*Note*: Numbers in italics indicate significant effects: italics with *** is significant with *p* < .001, italics significant with *p < .05 and bold italics is marginally significant. Two treatment levels ((i) Restricted and (ii) ample water levels), Cultivation status three levels ((i) wild, (ii) feral and (iii) cultivated), Genetic groups five levels ((i) Budongo, (ii) Itwara, (iii) Kibale, (iv) SC, and (v) Zoka), Location 7 levels ((i) Budongo, (ii) Itwara, (iii) Kalangala, (iv) Kibale, (v) Mabira, (vi) Malabigambo and (vii) Zoka).

Abbreviations: RGRA (d^−1^), Relative growth in leaf area; TNL, Total number of leaves; TLA (cm^2^), Total leaf area; TLDW (g), Total leaf dry weight; SLA (cm^2^ g^−1^), Specific Leaf area; RL (cm^3^ cm^−2^), Root volume to leaf area ratio.

**FIGURE 1:**
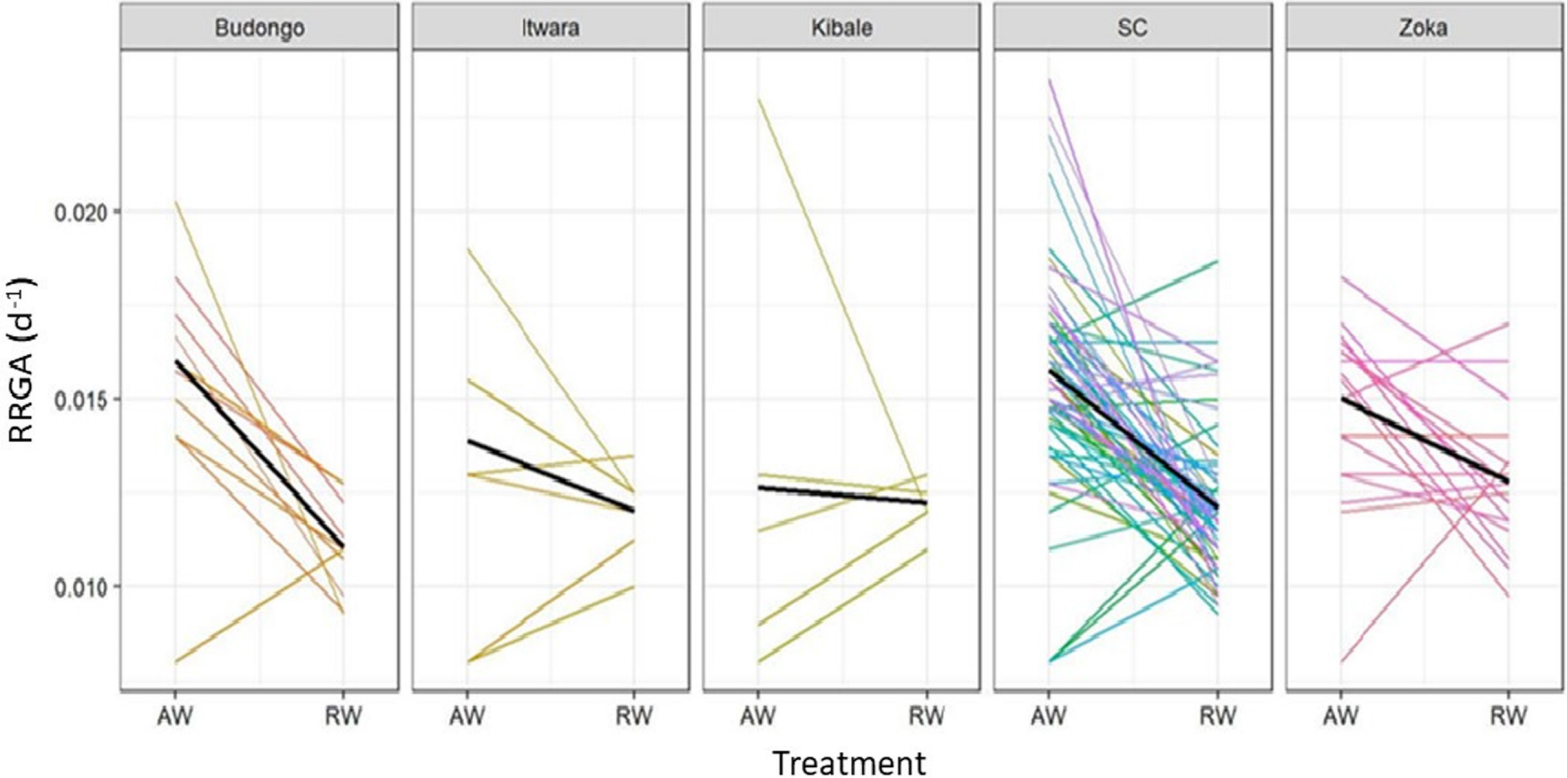
Mean RGRA (d^−1^) as a function of treatment (ample‐water (AW) and restricted‐water (RW) across genetic groups (panels) and genotypes (colored lines)). Solid black line shows the mean estimated response per genetic group

Genetic groups significantly differed in their TLA, TLDW and RL but not in the other three traits (Table 3). Plants from the genetic group SC had the highest mean TLA which was 60.9% higher than the lowest TLA observed in genetic group Kibale (Table S3). The effect of the genetic group on TLDW was similar to TLA with genetic group SC having 67.6% higher mean TLDW than genetic group Kibale which had the lowest TLDW (Table S3). For RL, Zoka had the highest value which was 43.9% higher than the lowest R_L_ observed in the genetic group Kibale (Table S3). Interaction effects between genetic groups and treatment were only observed in RGRA, implying that the magnitude of the response in this trait to the water treatment differed across genetic groups. The RGRA of genetic groups: Budongo, SC and Zoka were significantly reduced due to restricted‐water supply but not that of genetic groups Itwara and Kibale (Figure 1; Table S3).

Overall, location as a factor had stronger effects on growth response traits to ample and restricted‐water supply than the two other factors, cultivation status and genetic groups (Table 3). The location had significant main effects and interaction effects on all traits except TNL, TLDW and SLA (Table 3). This implies that the growth response values significantly differed depending on the location from which the genotypes were collected. For example, for TL, i.e., the response trait with the strongest location effects (Table 3), location Malabigambo had the highest average TLA which was 73.7% higher than the lowest TLA observed in Kibale. The water treatment had no significant effects on the TLA of genotypes collected from Zoka, Itwara, Kibale, Kituza and Kawanda, while it significantly reduced TLA of genotypes collected from Malabigambo, Kalangala and Mabira (Table S4). In absolute terms, under ample‐water conditions, Malabigambo had a significantly higher TLA (7263 (±153) cm^2^) than all other locations while Kibale's TLA (1413 (±38) cm^2^), was significantly lowers at all locations except Zoka (Table S4). Similarly, under restricted‐water conditions; Malabigambo had the highest TLA (3711 (±62) cm^2^) compared with all other locations, whereas Kibale had the lowest TLA (1469 (±42) cm^2^) which was significantly lower than T_L_ of all other locations except Zoka (Table S4).

### 3.3 Detailed effects of the experimental factors as illustrated with RGRA (our proxy trait for performance)

#### 3.3.1 RGRA across cultivation status: Wild, feral and cultivated

The relative effect of water treatment on RGRA was rather similar across cultivation status (Table 4; Figure S3A), hence confirming the finding in Table 4 (no significant main effect and interaction effects for cultivation status on RGRA). In absolute terms, under ample‐water conditions, wild genotypes had the highest RGRA which was significant, but only modestly, (5.7%) higher than the lowest RGRA, which was observed among the cultivated genotypes (Table 4; Figure S3A). Under restricted‐water treatment, wild genotypes still had the highest RGRA which was 5% higher than the lowest RGRA observed among feral genotypes (Table 4; Figure S3A).

**TABLE 4:** Mean values and standard error (SE) of relative growth rate in leaf area (RGRA (d^−1^)) of *C. canephora* subjected to ample and restricted water treatments

Factor	Ample‐water mean (SE)	Restricted‐water mean (SE)	Relative change (%)
Cultivation status			
Cultivated	0.0150 (0.0001) a	0.0120 (0.0001) a	−20.0
Feral	0.0156 (0.0001) a	0.0116 (0.0001) a	−25.6
Wild	0.0159 (0.0001) a	0.0122 (0.0001) a	−23.3
Genetic group			
Budongo	0.0163 (0.0001) a	0.0110 (0.0001) b	−32.5
Itwara	0.0144 (0.0001) a	0.0124 (0.0001) ab	−13.9
Kibale	0.0124 (0.0001) a	0.0120 (0.0001) ab	−3.2
SC	0.0159 (0.0001) a	0.0112 (0.0001) ab	−29.6
Zoka	0.0152 (0.0001) a	0.0125 (0.0001) a	−17.8
Location			
Budongo	0.0162 (0.0001) abc	0.0119 (0.0001) ab	−26.5
Itwara	0.0144 (0.0002) cd	0.0124 (0.0001) a	−13.9
Kalangala	0.0156 (0.0001) bc	0.0118 (0.0001) ab	−24.4
Kibale	0.0127 (0.0001) d	0.0118 (0.0001) ab	−7.1
Mabira	0.0175 (0.0002) a	0.0129 (0.0001) a	−26.3
Malabigambo	0.0169 (0.0002) ab	0.0107 (0.0001) b	−36.7
Zoka	0.0151 (0.0001) c	0.0126 (0.0001) a	−16.6

*Note*: Numbers are means, standard errors in brackets and different letters in the same column show significant differences among means at *p* < .05 of Relative growth rate (RGRA).

#### 3.3.2 RGRA across genetic groups

Table 4, Figures 1 and S3B show variation in the relative effect of restricted‐water on RGRA across genetic groups, with genetic group Budongo being the most strongly affected and genetic group Kibale being least affected (see also significant genetic group * treatment effect Table 3). Under ample‐water conditions, the absolute RGRA did not differ significantly between genetic groups while it did under restricted‐water conditions (Table 4, Figures 1 and S3B). Under restricted‐water conditions, genetic group Zoka had the highest RGRA which was 12.0% higher than the lowest RGRA observed for genotypes from genetic group Budongo (Table 4; Figure S3B). Additionally, Figure 1 and standard errors of means (Table 4) suggest that there was wider genotypic variation in RGRA across genetic groups under ample‐water conditions than there was under restricted‐water conditions.

#### 3.3.3 RGRA across locations

There was a large variation in the relative effect of water treatment on RGRA of genotypes collected from the different locations (Table 4; Figures S3C and S4). The effect of restricted‐water on RGRA was significant for all locations except for Kibale and Itwara (Tables 4, S5; Figure S4). The mean percentage change in performance was highest among genotypes collected from Malabigambo, Budongo, Mabira and Kalangala, respectively, while the effect of restricted‐water supply was smallest for genotypes collected from Kibale, Itwara, Zoka and Kituza, respectively (Table 4 and slope of the black lines in Figure S3). In absolute terms, under ample‐water conditions, genotypes from Mabira had a significantly higher mean RGRA, which was 27.4% higher than the lowest mean RGRA in location Kibale (Table 4; Figures S3C and S4). Similarly, in restricted‐water conditions, Mabira had the highest and Kibale had the lowest RGRA but the difference was much smaller (8.5%) (Table 4; Figures S3C and S4). Therefore, differences between locations tended to converge in the restricted‐water treatment.

Across the studied experimental factors (cultivation status, genetic group and location), it is worth noting that results showed a tendency of some genotypes to have higher RGRA under restricted‐water conditions than with ample‐water although this effect was not statistically significant in any of these cases (*p* > 0.05) (Figures 1 and S4). The effect occurred in genotypes with both high and low RGRA values in the ample‐water treatment and therefore are very unlikely an experimental artifact, whereby the genotypes could not have been adequately watered under ample‐water conditions. Additionally, for some genotypes, the effect could be due to variations in sample size causing the mean in restricted‐water to be higher than that under ample‐water conditions.

### 3.4 Multivariate analysis of growth‐related traits

**FIGURE 2:**
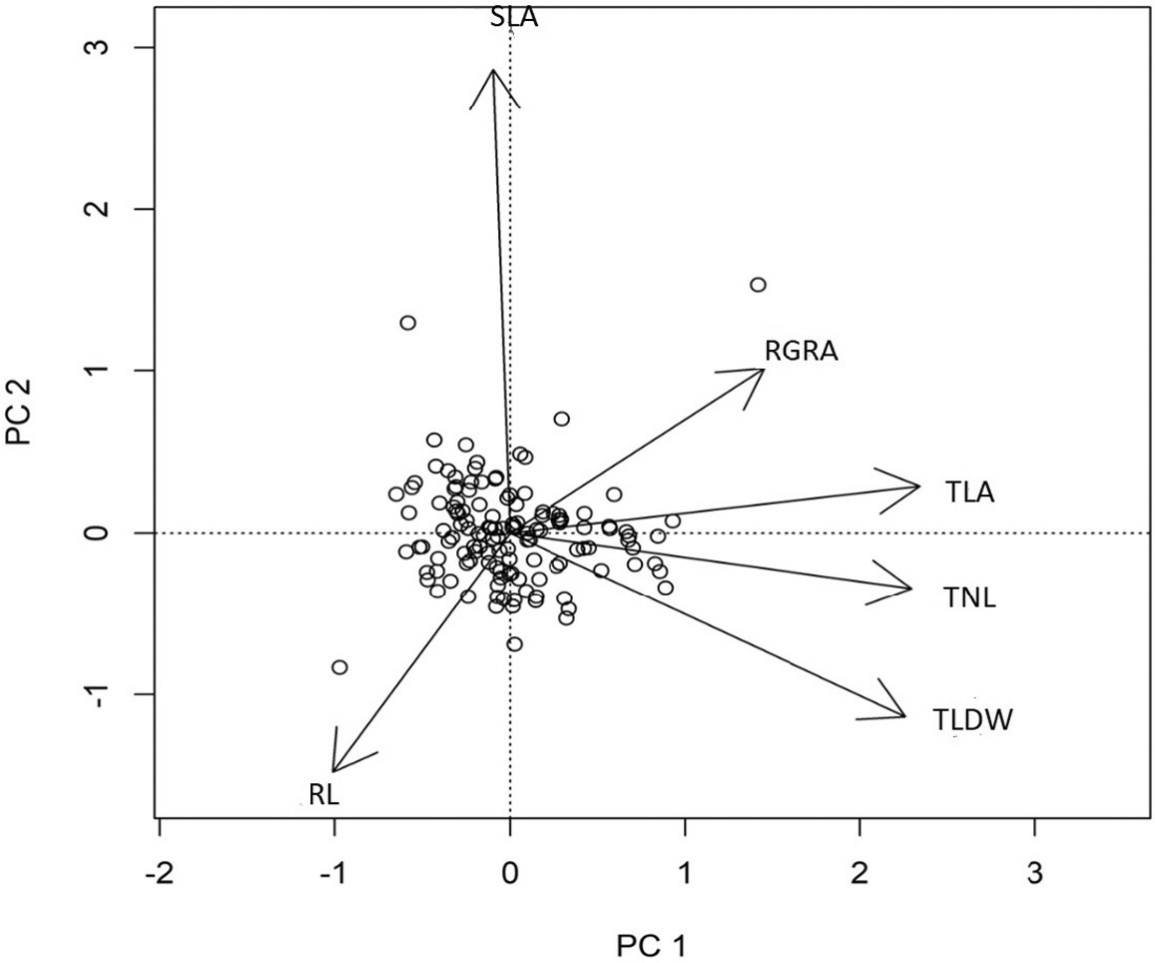
Principal component analysis (PCA) of genotypic mean trait values showing multivariate dependency between the measured traits: Relative growth in leaf area (meanRGRA), Total number of leaves (mean TLA), Total leaf area (meanTNL), Total leaf dry weight (meanTLDW), specific leaf area (meanSLA), root volume to leaf area ratio (meanRL). The two first axis, PC1 and PC2, account for 46% and 20% of the total variation respectively.

The PCA analysis showed that TNL, TLA and TLDW were most loaded on the first PCA axis (explaining 46% of the variation), while SLA was mostly loaded on the second PCA axis (explaining 20% of the variation). See Figure 2. The PCA on the individual replicates showed a similar pattern. Therefore, SLA varied mostly independently of TLA (correlation −0.002). The PERMANOVA showed that treatment, location and cultivation status significantly affected the dissimilarities between genotypes (*p*‐values respectively <.001, .03, <.001) see Table S6. Treatment explained 20% of the variation in the traits, location 10% and cultivation status only 1.8%.

### 3.5 What is the relationship between performance in ample and restricted‐water conditions?

**FIGURE 3:**
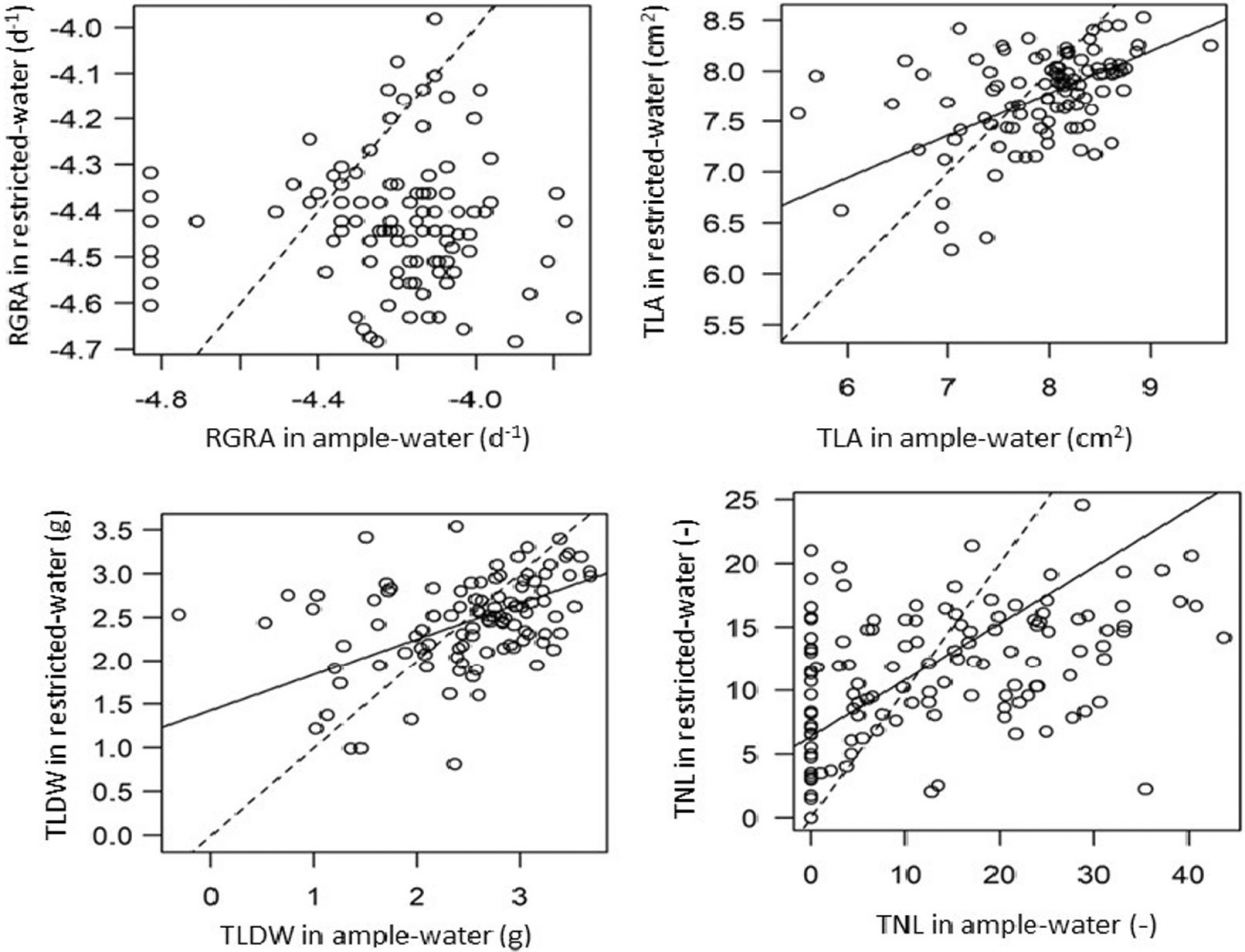
Relationship between growth‐related traits in ample water versus restricted water. The relationships were fitted with type II regression. Plots with solid black lines show relationships that were significantly different from zero. For reference, 1:1 line is shown (dotted line).

The type II regression where the genotypic means of growth‐related traits were regressed to each other in ample‐water versus restricted water revealed that across the four traits, the genotypes that performed well in ample water performed relatively less well in restricted water. In all cases of the aforementioned regressions, the slope was less than one. For RGRA there was no significant relationship between the values in ample water and those in restricted water suggesting that comparatively well‐performing genotypes are strongly compromised in restricted water. See Figure 3 and Table S7. for a table with statistics.

### 3.6 What is the relationship between performance and tolerance under restricted‐water conditions and the wetness index of locations?

**FIGURE 4:**
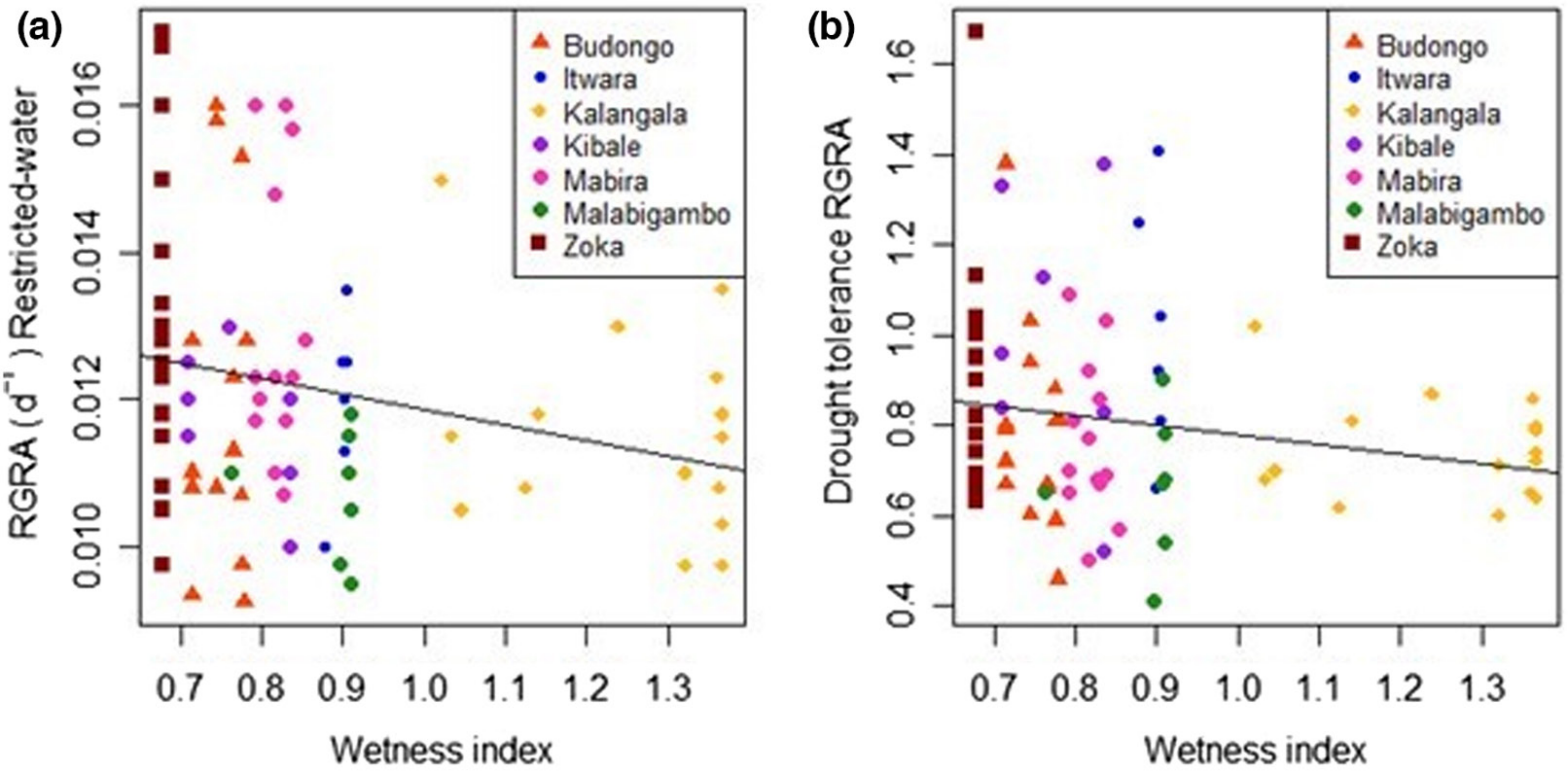
Relationship between the performance of *C. canephora* genotypes in restricted‐water conditions and wetness index of the location in which they were collected from (a) and the relationship between drought tolerance as estimated from RGRA and wetness index of the location (b); wetness index (WI), high WI values indicate moist conditions and low WI values indicate dry conditions. Both slopes were negative and significantly different from zero at *p* = .05.

There was a significantly (*p* = .03, *R* ^2^ = .06) negative relationship between RGR_A_ of genotypes in restricted‐water conditions and the wetness index of the climate of a genotype's origin (Figure 4a), illustrating that genotypes from relatively wet areas (high wetness index) tended to have lower RGRA in the restricted‐water treatment than those from drier locations. Performance of a genotype in restricted‐water conditions could partially be predicted from the wetness index of its geographic location by the following formula: RGRA_restricted‐water_ (d^−1^) = 0.014–0.002 (wetness index), SE = 0.001, *R* ^2^ = 0.06, *F*(1, 80) = 4.85, *p* = .03. The fitted slope (−0.002) has a confidence interval of (−0.0040, −0.0002) at 95.5% implying that performance under restricted‐water conditions is negatively correlated with WI.

There was also a marginally significant negative (*p* = .05) relationship between drought tolerance of genotypes and wetness index in their location (Figure 4b), being defined by: Tolerance = 0.99–0.214 (wetness index), *R* ^2^ = 0.05 and SE = 0.106. The negative relationship between tolerance and wetness index of the locations possibly indicates a climatic signature related to drought tolerance of the genotypes. This observed relationship between drought tolerance and wetness index suggests that on average, genotypes from the comparatively drier areas, e.g., Zoka, tended to be somewhat more drought tolerant than genotypes from the wetter area Kalangala. No difference in terms of goodness of fit was found between the linear and the other two types of non‐linear models. Other traits also tended to show a similar trend of drought tolerance being negatively correlated with the wetness index although statistically significant relations were observed in TNL only (Figure S5 and Box S2).

## 4 DISCUSSION

In this study, we explored Uganda's *C. canephora* genotypic diversity in a screening experiment with 148 genotypes. We specifically explored: (i) the effects of water treatment on growth categorized by cultivation status (wild, feral and cultivated), genetic groups and the geographic location, (ii) the relationship between performance under restricted‐water and performance under ample‐water conditions and (iii) the relationship between drought tolerance and wetness index (WI). To our knowledge, this is the first study to explore intra‐specific variation in responses to water treatment for a large number of genotypes (>100) in tropical tree species.

### 4.1 Effect of water treatment on *C. canephora* in growth response traits

Water treatment significantly reduced the RGRA (relative growth rate of leaf area), TNL (total number of leaves), TLA (total leaf area), TLDW (total leaf dry weight), SLA (specific leaf area) and increased the RL (root volume to leaf area) (Table 2). The latter finding concurs with the optimal partitioning theory which entails that in response to stress, plants allocate proportionally more resources to the structure capturing the most limiting resources (Bloom et al., 1985; Brouwer, 1963). Other studies (Ryser & Eek, 2000; Shipley & Meziane, 2002) and reviews (Eziz et al., 2017; Hoffmann & Poorter, 2002) also stated that, in response to stress, plants adjust their biomass allocation in accordance to whether the most limiting resource is above‐ or below ground. In our study, TNL and TLA were the most affected traits (Table 2) implying that genotypes responded to restricted‐water mainly by minimizing transpirational water loss by reducing the number of leaves and leaf area. Differential reduction in leaf area as a response to drought stress has also been observed by other authors (DaMatta et al., 2003; Dias et al., 2007; King'oro, 2014; Pinheiro et al., 2004). Our current findings extend these observations to a wider range of genotypes including wild, feral and cultivated material.

### 4.2 Variation in response across cultivation status, genetic groups and location

Our findings indicate that there is a clear genotypic variation in performance (RGRA) both under ample and restricted‐water conditions (Figures 1 and S4). The variation in RGRA was larger (more than two‐fold) under ample‐water than restricted‐water conditions (Table 3; Figures 1 and S4). The different phenotypic responses of genotypes in ample and restricted‐water conditions (Figures 1 and S4) probably reflects an underlying genetic polymorphism that may drive different phenotypic responses to different environments (Forsman, 2015; Pigliucci, 2005; Stearns, 1989). The observed genotypic variation in our study in both growth and drought tolerance can be utilized for optimizing breeding programs initiatives to develop drought‐tolerant varieties with adequate yield capacity (Table 3; Figures 1 and S4). Results did not show significant variations in RGRA between genotypes of different cultivation status (wild, feral or cultivated). This probably indicates that Uganda's breeding efforts to date have not addressed drought tolerance. Breeding efforts have been focusing on other factors, e.g., yield and resistance to pests and diseases, in particular generating wilt disease‐resistant coffee varieties (Musoli et al., 2008). Breeding efforts in *C. canephora* are relatively limited, partially due to the perennial nature of the crop (with an economic lifespan of about 20 years), which suggests that most of the cultivated material is still very similar to the wild genotypes (Montagnon et al., 1998; Ngugi & Aluka, 2019; Thomas, 1935). Indeed, Kiwuka et al. (2021) found that Uganda's cultivated genotypes were genetically similar to wild populations from Malabigambo, Mabira and Kalangala forests.

Across the experimental factors we studied, location exhibited the widest range of reductions in RGRA from 7.1% to 36.7% in Kibale and Malabigambo respectively (Table 4; Figure S3). The genetic distinctiveness of Uganda's wild *C. canephora* populations across locations as shown in Kiwuka et al. (2021) (Table S1) and their differential phenotypic response to drought (Table 4; Figure S4) indicate that Uganda's *C. canephora* diversity could be locally adapted to the climatic conditions within the locations. The significant interaction effect between genetic group and water treatment (Table 3) also provides evidence that the localization of the genetic groups (i.e., Zoka, Itwara, Kibale and Budongo genetic groups from the NW) could be associated with genetic effects and putatively to adaptive potential. The possibility of genotypes being locally adapted is also indicated by an overlap between the genetic group (Figure 1) and location (Figure S4) effects on RGRA. However, the strong effect of location on response to restricted water could also be reflecting local differences in other factors such as soil types that may influence selection for the difference in growth‐related traits.

### 4.3 Slow growth as a strategy to cope with drought stress and evidence of a trade‐off between growth and drought tolerance

Genotypes that had low RGRA, TNL, TLA and TLDW values in ample‐water conditions were comparatively less affected by restricted‐water, a scenario which indicates a trade‐off between growth and drought tolerance across the study populations (Figure 3). This finding concurs with an established ecological paradigm that there is a trade‐off between the capacity of plants to grow fast when resources are abundant and their capacity to tolerate resource shortages (Aerts & Chapin, 1999; Bazzaz & Bazzaz, 1996; Grime, 2006). The trade‐off between growth and tolerance has been linked to a conservative resource‐use strategy in which slow growth results in slow tissue turnover (i.e., conservative use of resources) and subsequently less dependency on the environment for the acquisition of new resources. On the contrary fast growth is associated with high resource turnover rates, intensive resource acquisition, high dependency on the environment and ultimately shorter lifespan (Chapin, 1980; Chapin III et al., 1993; Grime et al., 1997; Reich et al., 2003; Sterck et al., 2006, 2011). Ecologically, slow growth has been reported as an adaptive strategy for plants in resource‐limiting conditions. Poorter (1989) studied the ecological consequences of the interspecific variation in the relative growth rate (RGR) of plants and concluded that differences in potential RGR between species were habitat‐related whereby fast‐growing species were found in resource‐rich habitats while slow growers could be found in any adverse environmental condition. In response to restricted‐water, a growth‐tolerance trade‐off could be expected because several traits and mechanisms that confer tolerance in dry conditions (e.g., low specific leaf area, low stomatal size or number) reduce water loss but also reduce rates of net photosynthesis per unit area, which, in turn, results into slower growth under favorable water availability (Lambers et al., 2008; Sterck et al., 2011).

Although the growth‐tolerance trade‐off has been widely studied and established across species (interspecific), including tropical forest trees (Amissah et al., 2018; Poorter & Jong, 1999; Sterck et al., 2006, 2011) much fewer studies (Menezes‐Silva et al., 2015; Pallardy & Kozlowski, 1981; Silva et al., 2013) have been conducted to explore the intraspecific variation of tropical trees to drought and the manifestation of the growth‐tolerance trade‐off. Pallardy and Kozlowski (1981) revealed a probable growth‐tolerance trade‐off among *Populus* clones: fast‐growing clones had a larger initial rate of decline in leaf water potential with transpirational flux density but reduced the rate of decline more than slow‐growing clones as the transpirational flux density increased. Similarly, Menezes‐Silva et al. (2015) and Silva et al. (2013) studied eight clones of cultivated *C. canephora* (variety Conilon) and found that wood density, a trait that partially influences the plant's water‐conducting capacity, was higher in drought‐tolerant clones, and was associated with greater resistance to cavitation. This adaptation however could limit growth under favorable water conditions as dense wood is more costly to produce and the associated smaller xylem have lower maximum water conductance (Menezes‐Silva et al., 2015; Silva et al., 2013).

In our study, the relatively low RGR_A_ and high tolerance of genotypes from Kibale, Itwara and Zoka locations (Table 4; Figures S3C and S4) suggests that those populations employ a more conservative resource‐use strategy, while genotypes from Mabira, Malabigambo, Kalangala and Budongo employ a more rapid resource‐acquisition strategy. Similar to our results, Silva et al. (2013) and Menezes‐Silva et al. (2015) also found that across a set of cultivated *C. canephora* clones, the most drought‐tolerant ones tended to be slow growers. Slow growth in stressful conditions could in the long term be more adaptive than fast growth because fast growth results in larger and more resource‐demanding plants that could eventually die off if the resource demand is not met. Here, we showed the existence of a growth‐tolerance trade‐off across a large set of wild accessions of a perennial crop species, suggesting that intraspecific variation in tolerance may be related to selection in natural environments. Evidence of a growth‐tolerance trade‐off in our study is further corroborated in our related experiment by Kiwuka (2020) where we studied fewer (15) genotypes with more response traits and found that slow‐growing genotypes were more drought tolerant and less plastic for most of the response traits.

In interpreting our findings, it should be noted that our experiment was a pot experiment and pots have limited volume. Firstly, this could cause a so‐called pot‐binding effect (Poorter et al., 2012; Sinclair et al., 2017); pots holding insufficient water to support transpiration and therefore growth. This could be more severe for fast‐growing plants than for slow‐growing ones. However, in our set up we accounted for this effect as we determined the relationship between water consumption and plant size and adjusted the amount of water gift in restricted water treatment to correct for larger plants consuming more water (see Section 2.4). Therefore, we are confident that larger plants did not suffer greater drought stress than smaller ones in the water‐restricted treatment and that the pot‐binding effect was minimized as seen in Plate S1 and S2. Secondly, in the field, rooting depth can be a drought‐adaptive trait as it allows access to deeper moister soil layers. This effect evidently could not be mimicked in pots. The association of rooting depth with growth potential could be either positive (fast growth facilitating deeper roots) or negative (larger deeper root systems imposing greater metabolic costs and therefore, slowing growth). Altogether, it is important to determine whether the drought‐tolerance trade‐off found in our study also occurs in the field. If the observed growth‐tolerance trade‐off occurs under field conditions, it would pose a dilemma for breeding on what to select if one cannot have both. For instance, selecting fast growth could result in low drought tolerance which poses a challenge, especially for small‐scale coffee farmers who may not have irrigation facilities to deal with drought spells. Therefore, to sustain *C. canephora* production in drought‐prone environments, breeders should break the negative correlation between poor performance and tolerance (Table 4, Figure 3). This proposition agrees with DaMatta (2018) who suggested that breeding for drought tolerance in coffee should aim at developing tolerant genotypes with “acceptable yields”. Despite the adaptive advantage of slow growth (conservative resource‐use strategy), its positive association with low performance is also a challenge as farmers are interested in good yields. Selection for either slow or fast‐growing genotypes should therefore be done in consideration of whether the intended product is for stressful or optimal conditions.

### 4.4 The link between drought tolerance and local climate

Our results indicated a weak but statistically significant climatic signal concerning to drought tolerance (Figure 4). There appears to be a trend where genotypes from wetter locations (higher wetness index, WI) tended to be less drought tolerant than those from drier ones (lower WI) (Figures 4 and S4). Our findings, therefore, seem to agree with our expectation, i.e., that genotypes from drier areas would be more drought tolerant than genotypes from wetter areas, though the low *R* ^2^ of the relationship indicates that the observed signal is not very strong. These results concur with Choat et al. (2007) who observed that differences in water availability across sites could drive intraspecific variation among *Cordia* species. Studies (Baquedano et al., 2008; Bongarten & Teskey, 1986; Peuke et al., 2002) documented that the ecotypes of *Pinus taeda*, *Fagus sylvatica*, *Quercus coccifera*, had adaptive features which were probably driven by the local climate. In our results, WI explained approximately 5% of the variation in drought tolerance in RGRA across genotypes and further analysis preferably over a wider climate range as well as WI data obtained from higher resolution weather data is needed to verify the consistency of this trend. Next, other factors may affect drought tolerance such as soil hydraulic properties and local topology. Finally, drought tolerance as determined in our study experiment may not fully reflect drought stress in the field (see next section).

### 4.5 Considerations regarding the experimental set‐up

This paper presented results from a large screening experiment where 148 genotypes comprising 61% wild, 7% feral and 32% cultivated, were subjected to modest drought (restricted‐water) and ample‐water regimes (see Table S3). As such, for the feasibility of the experiment, we included maximally four replicates per genotype per treatment because this was the maximum manageable number, allowing for the identification of the largest differences within the material. Damage and mortality of some plants caused variation in the real number of replicates across genotypes (Table S8). Consequently, the mixed‐effects model that we applied could not estimate genotype effects very precisely but, rather, it put the genotype effects closer to the mean effect (an effect called shrinkage). It is therefore important to note that in our analyses, individual genotypes acted mostly as a replication at the genotypic level to test cultivation status, genetic group and location effects on the responses.

Despite the close relationship between vegetative growth and yield capacity of coffee plants (Cilas et al., 2006), one should note that our study focused on responses of comparatively juvenile plants and we did not include effects of ontogenetic changes on responses yet certain ontogenetic changes may affect performance in later life stages. For example, as mentioned above in the discussion about growth‐tolerance trade‐offs, relatively fast growth in young plants under dry conditions, could be maladaptive later in life as it can result in larger more water‐demanding phenotype. To assess how drought affects trees over a larger time of their life, more mature trees (of about 5 years) need to be considered.

### 4.6 Conclusion and implications

Considering climate change and its adverse effects on coffee production, this study showed that Uganda has potentially adapted *C. canephora* genetic diversity which could be used to develop drought‐tolerant genotypes. Breeders, however, need to work toward weakening or even breaking the trade‐off between drought tolerance and performance. As noted by Borrell et al. (2020) the conservation of extant genetic diversity, particularly in a period of rapid environmental change is critical to support future crop improvement. In this regard, the Zoka population is of special interest among the whole *C. canephora* natural distribution in Africa, being within the drier end of the climatic gradient and exhibiting relatively high drought tolerance. Zoka is a small unique forest (the only tropical rainforest occurring in dry northern Uganda), but its small size (12.6 km^2^) makes the population particularly vulnerable to habitat destruction. At a national level, there is a need to foster the in‐situ conservation and management of Uganda's *C. canephora* wild populations. Strategic in‐situ conservation of these wild populations will allow for their evolution and adaptation to environmental stresses and consequently the continued use of the material to offer resilience to cultivated *C. canephora* material amidst the escalating effects of climate change. National conservation strategies should involve the restriction of *C. canephora* cultivation near any wild population to deter genetic drift and allow continuous adaptation of the natural populations.

## AUTHOR CONTRIBUTIONS

**Catherine Kiwuka:** Conceptualization (equal); data curation (lead); formal analysis (equal); funding acquisition (lead); investigation (equal); methodology (equal); project administration (lead); resources (equal); software (equal); validation (equal); visualization (equal); writing – original draft (lead); writing – review and editing (equal). **Jan Vos:** Conceptualization (equal); data curation (equal); formal analysis (equal); funding acquisition (equal); investigation (equal); methodology (equal); project administration (equal); resources (equal); software (equal); supervision (lead); validation (equal); visualization (equal); writing – original draft (equal); writing – review and editing (equal). **Jacob C. Douma:** Conceptualization (equal); data curation (equal); formal analysis (lead); investigation (equal); methodology (equal); resources (equal); software (equal); supervision (supporting); validation (equal); visualization (equal); writing – review and editing (equal). **Pascal Musoli:** Conceptualization (equal); data curation (equal); formal analysis (supporting); funding acquisition (equal); investigation (equal); methodology (equal); project administration (supporting); resources (equal); supervision (equal); writing – review and editing (equal). **John W. Mulumba:** Conceptualization (equal); data curation (equal); formal analysis (supporting); funding acquisition (equal); investigation (equal); methodology (equal); project administration (supporting); resources (equal); supervision (supporting); validation (equal); visualization (equal); writing – review and editing (equal). **Valerie Poncet:** Conceptualization (equal); data curation (equal); formal analysis (equal); funding acquisition (equal); investigation (equal); methodology (equal); project administration (supporting); resources (equal); software (equal); supervision (equal); validation (equal); visualization (equal); writing – review and editing (equal). **Niels P. R. Anten:** Conceptualization (equal); data curation (equal); formal analysis (equal); funding acquisition (equal); investigation (equal); methodology (equal); project administration (equal); resources (lead); software (equal); supervision (lead); validation (lead); visualization (equal); writing – review and editing (equal).

## FUNDING INFORMATION

The activities of this study were mainly funded by the World Bank‐supported project entitled “Agricultural Technology and Agribusiness Advisory Services” (ATAAS) through National Agricultural Research Organization (NARO). Additionally, we thank the Uganda Coffee Development Authority (UCDA) and the Centre for Crop Systems Analysis (CSA) of the Production Ecology and Resource Conservation Graduate School of Wageningen University and Research for additional financial assistance to support the experimental phase of the study.

## CONFLICT OF INTEREST

None declared.

## Supporting information


Appendix S1

## Data Availability

Data associated with this manuscript is included and will be archived in the Dryad data repository.
