# Supplementary material for: Intraspecific variation in growth response to drought stress across geographic locations and genetic groups in Coffea canephora
Source: Ecol Evol. 2023 Jan 3;13(1):e9715. doi: 10.1002/ece3.9715 (PMC9810788; doi:10.1002/ece3.9715)
Supplement: Supplementary file 1 — Appendix S1 [file ECE3-13-e9715-s001.docx]

APPENDICES

Appendix A

| Appendix Table A.1. Number of genotypes included in the study per genetic group per location | | | | | |
| --- | --- | --- | --- | --- | --- |
| **Location** | **Genetic groups** | | | | |
|  | **SC** | **NW** | | | |
|  |  | Itwara | Kibale | Budongo | Zoka |
| Budongo |  |  |  | 11 |  |
| Itwara |  | 10 |  |  |  |
| Kalangala | 18 |  |  |  |  |
| Kawanda | 14 |  |  |  |  |
| Kibale |  |  | 7 |  |  |
| Kituza | 24 |  |  |  |  |
| Mabira | 13 |  |  |  |  |
| Malabigambo | 7 |  |  |  |  |
| Zoka |  |  |  |  | 23 |
| *This genetic grouping is as per Kiwuka et al.* (Kiwuka *et al.*, 2021)*. SC (Southern Central), NW (North Western; which differentiates into four other distinct groups namely: Itwara, Kibale, Budongo and Zoka). Note: genotypes that were misclassified and/or hybrids were not considered* | | | | | |

| Appendix Table A. 2. Traits measured to investigate response to water treatment | | |
| --- | --- | --- |
| Collection phase | Trait | Units |
| Start of the treatment (25^th^ May 2017) | plant height | cm |
|  | leaf area | cm^2^ |
|  | no. of main stem leaves |  |
|  | stem diameter 5 cm for above the mark,  6 cm for below the mark | mm |
| During treatment  (21^th^ - 24^th^ June 2017) | plant height | cm |
|  | leaf area | cm^2^ |
|  | no. of primaries |  |
|  | no. of suckers |  |
|  | no. of leaves on main stem, primaries and suckers |  |
|  |  |  |
| End of Treatment (12^th^ -26^th^ September 2017) | plant height, | cm |
|  | leaf area | cm^2^ |
|  | no. of primaries |  |
|  | no. of suckers |  |
|  | no. of leaves on the main stem, primaries and suckers |  |
|  | fresh weight of all leaves | g |
|  | dry weight of all leaves | g |
|  | specific leaf area | cm^2^ g^-1^ |
|  | root volume | cm^3^ |

| *Appendix Table A.3. Growth variables of C. canephora subjected to ample-water (AW) and restricted-water (RW) treatments: total number (TNL); total leaf area (TLA [cm^2^]); total leaf dry weight (TLDW [g]); specific leaf area (SLA [cm^-2^ g^-1^]); root volume to total leaf area (RL [cm^3^ cm^-2^]). Data collected at the end of the experiment* | | | | | | | | | | | | | | | | | | | |
| --- | --- | --- | --- | --- | --- | --- | --- | --- | --- | --- | --- | --- | --- | --- | --- | --- | --- | --- | --- |
| Category | TNL | | | | TLA | | TLDW | | | | SLA | | | | | | RL | | |
| *Cultivation Status* | AW | | RW | | AW | RW | AW | | RW | | AW | | | RW | | | AW | RW | |
| Cultivated | 13(0.4) a | 12(0.2) a | | 3214(61) a | | 2635(45) a | | 16(0.4) a | 14(0.3) a | | 250(5) a | | | 220(3) ab | | | 0.0081(0.0003) a | | 0.0093 (0.0002) a |
| Feral | 15(0.5) a | 14(0.2) a | | 3941(59) a | | 3024(47) a | | 21(0.4) a | 17(0.3) a | | 211(2) a | | | 197(2) b | | | 0.0057(0.0001) b | | 0.0080 (0.0001) a |
| Wild | 13(0.4) a | 11(0.3) b | | 3807(88) a | | 2285(47) b | | 17(0.4) a | 12(0.3) b | | 255(3) a | | | 232(3) a | | | 0.0062 (0.0001) b | | 0.0091 (0.0003) a |
| *Genetic groups* | AW | RW | | AW | | RW | | AW | RW | | AW | | | RW | | | AW | | RW |
| Budongo | 16(0.5) a | 9(0.3) bc | | 3372(56) ab | | 2245(46) ab | | 16(0.3) ab | | 12(0.3) ab | | | 228(2)a | | 221(3) ab | 0.0068(0.0003) a | | | 0.0074(0.0001) ab |
| Itwara | 7(0.5) b | 9(0.3) bc | | 3778(105) ab | | 2377(60) ab | | 17(0.5) ab | | 12(0.3) ab | | | 275(4)a | | 230(2) ab | 0.0060(0.0001) a | | | 0.0080(0.0001) ab |
| Kibale | 3(0.2) b | 6(0.2) c | | 1519(38) b | | 1188(29) c | | 6(0.2) b | | 5(0.2) c | | | 288(4)a | | 258(3) a | 0.0050(0.0001) a | | | 0.0046(0.0001) b |
| SC | 16(0.5) a | 13(0.2) a | | 416492) a | | 2763(51) a | | 19(0.4) a | | 15(0.3) a | | | 247(5)a | | 216(3) ab | 0.0068(0.0002) a | | | 0.0094(0.0002) a |
| Zoka | 9(0.4) b | 11(0.3) ab | | 2472(59) b | | 1993(46)b | | 11(0.3) b | | 10(0.3) bc | | | 266(4)a | | 248(5) a | 0.0063(0.0001) a | | | 0.0108(0.0005) a |
| *Location* | AW | RW | | AW | | RW | | AW | RW | | AW | | | RW | | | AW | | RW |
| Budongo | 16(0.6) b | 9(0.3) cd | | 3251(70) cd | | 2177(50) c | | 16(0.4) cd | | 12(0.4) b | | 231(2) b | | 220(3) ab | | | 0.0066(0.0003) a | | 0.0079(0.0002) ab |
| Itwara | 7(0.5) cd | 9(0.4) cd | | 3778(117) bcd | | 2377(67) bc | | 17(0.6) bcd | | 12(0.4) b | | 275(5) ab | | 230(3) ab | | | 0.0060(0.0001) a | | 0.0080(0.0001) ab |
| Kalangala | 17(0.6) b | 13(0.3) ab | | 4381(89) b | | 3011(53) b | | 22(0.5) ab | | 17(0.4) a | | 212(2) b | | 200(2) b | | | 0.0059(0.0001) a | | 0.0086(0.0002) ab |
| Kibale | 4(0.2) d | 8(0.3) | | 1413(38) e | | 1469(42) d | | 5(0.2) e | | 6(0.2) c | | 319(5) a | | 258(3) a | | | 0.0062(0.0001) a | | 0.0052(0.0001) b |
| Mabira | 16(0.5) b | 12(0.3) bc | | 4110(92) bc | | 2293(49) c | | 19(0.5) bc | | 11(0.3) b | | 230(2) b | | 212(2) ab | | | 0.0066(0.0001) a | | 0.0107 (0.0002) a |
| Malabigambo | 30(0.4) a | 16(0.3) a | | 7263(153) a | | 3711(62) a | | 27(0.5) a | | 18(0.4) a | | 309(7) a | | 221 (2) ab | | | 0.0053(0.0001) a | | 0.0084(0.0002) ab |
| Zoka | 10(0.5) c | 11(0.3) bcd | | 2478(65) de | | 1921(51) cd | | 11(0.3) de | | 9(0.3) bc | | 266(4) ab | | 256 (6) a | | | 0.0059(0.0002) a | | 0.0103(0.0006) a |
| *Numbers are means, standard errors in brackets and different letters within a column show significantly different means at p < 0.05* | | | | | | | | | | | | | | | | | | | |

| Appendix Table A.4. Details of the effect of treatment and location on total leaf area (TLA) | | | | | | |
| --- | --- | --- | --- | --- | --- | --- |
| Factor | Value | | Std. Error | DF | t-value | p-value |
| Location**:** trt | | | | | | |
| Budongo**:** trt | | -1025 | 360 | 708 | -2.8487 | 0.005 |
| Itwara: trt | | -842 | 957 | 708 | -0.8803 | 0.379 |
| Kalangala: trt | | -1443 | 401 | 708 | -3.5999 | 0.000 |
| Kibale: trt | | 229 | 484 | 708 | 0.4744 | 0.635 |
| Mabira: trt | | -1665 | 466 | 708 | -3.5709 | 0.000 |
| Malabigambo: trt | | -3552 | 825 | 708 | -4.3070 | 0.000 |
| Zoka: trt | | -500 | 318 | 708 | -1.5756 | 0.116 |
| *trt denotes experimental treatment, experiment treatments had a significant effect when p < 0.05* | | | | | | |

| Appendix Table A.5. Numbers of plant individuals per treatment across cultivation status domestication | | | |
| --- | --- | --- | --- |
|  | Treatment | | Total |
|  | Ample-water | Restricted-water |  |
| Cultivated | 121 | 178 | 299 |
| Feral | 28 | 41 | 69 |
| Wild | 225 | 326 | 551 |
| Grand total | 374 | 545 | 919 |

| Appendix Table A.6. Number of replicates per treatment per genotype | | | | |
| --- | --- | --- | --- | --- |
| Location | Genotype | Number of replicates per treatment | | Total |
|  |  | Ample-water | Restricted-water |  |
| Budongo | BD 1.1 | 4 | 4 | 8 |
|  | BD 1.5 | 4 | 3 | 7 |
|  | BD 2.1 | 2 | 4 | 6 |
|  | BD 2.2 | 3 | 4 | 7 |
|  | BD 2.3 | 4 | 4 | 8 |
|  | BD 2.4 | 4 | 4 | 8 |
|  | BD 2.5 | 4 | 3 | 7 |
|  | BD 3.1 |  | 2 | 2 |
|  | BD 3.2 | 4 | 4 | 8 |
|  | BD 3.3 | 3 | 4 | 7 |
|  | BD 4.1 | 4 | 3 | 7 |
|  | BD 4.2 | 2 | 4 | 6 |
|  | BD 4.3 | 2 | 4 | 6 |
|  | BD 4.4 | 4 | 4 | 8 |
|  | BD 4.5 | 1 | 4 | 5 |
|  | BD 5.5 | 4 | 4 | 8 |
| Itwara | IT 2.2 | 1 | 4 | 5 |
|  | IT 2.3 | 3 | 4 | 7 |
|  | IT 3.3 | 3 | 4 | 7 |
|  | IT 4.2 | 4 | 4 | 8 |
|  | IT 4.3 | 1 | 4 | 5 |
|  | IT 4.4 |  | 2 | 2 |
|  | IT 4.5 |  | 4 | 4 |
|  | IT 5.1 |  | 1 | 1 |
|  | IT 5.2 |  | 1 | 1 |
|  | IT 5.3 | 1 | 3 | 4 |
| Kibale | KB 2.1 | 4 | 4 | 8 |
|  | KB 2.2 |  | 2 | 2 |
|  | KB 2.4 |  | 4 | 4 |
|  | KB 3.1 | 1 | 3 | 4 |
|  | KB 3.3 | 3 | 4 | 7 |
|  | KB 3.4 | 2 | 4 | 6 |
|  | KB 4.3 | 1 | 2 | 3 |
|  | KB 4.4 | 1 | 3 | 4 |
|  | KB 4.5 | 1 | 2 | 3 |
| Kalangala | KL 1.1 | 3 | 4 | 7 |
|  | KL 1.2 | 2 | 4 | 6 |
|  | KL 1.3 | 4 | 4 | 8 |
|  | KL 1.4 | 4 | 4 | 8 |
|  | KL 1.5 | 4 | 4 | 8 |
|  | KL 2.2 | 4 | 4 | 8 |
|  | KL 2.4 | 2 | 4 | 6 |
|  | KL 3.2 | 4 | 3 | 7 |
|  | KL 3.5 | 4 | 4 | 8 |
|  | KL 4.3 | 4 | 4 | 8 |
|  | KL 5.2 | 1 | 3 | 4 |
|  | KL 5.3 |  | 2 | 2 |
|  | KL 5.4 | 3 | 4 | 7 |
|  | KL 6.1 |  | 4 | 4 |
|  | KL 6.2 | 4 | 4 | 8 |
|  | KL 6.3 | 4 | 4 | 8 |
|  | KL 6.4 | 1 | 4 | 5 |
|  | KL 7.2 | 1 | 4 | 5 |
|  | KL 8.3 |  | 2 | 2 |
| Kituza | KT 0.1 | 1 | 4 | 5 |
|  | KT 0.2 | 3 | 4 | 7 |
|  | KT 0.3 | 1 | 3 | 4 |
|  | KT 0.4 | 2 | 3 | 5 |
|  | KT 0.5 | 4 | 4 | 8 |
|  | KT 0.7 | 4 | 4 | 8 |
|  | KT 0.8 | 2 | 4 | 6 |
|  | KT 1.0 |  | 3 | 3 |
|  | KT 1.2 | 3 | 4 | 7 |
|  | KT 1.6 | 3 | 4 | 7 |
|  | KT 1.7 | 4 | 4 | 8 |
|  | KT 1.8 | 2 | 4 | 6 |
|  | KT 2.0 | 1 | 4 | 5 |
|  | KT 2.1 |  | 4 | 4 |
|  | KT 2.3 | 2 | 4 | 6 |
|  | KT 2.4 | 3 | 4 | 7 |
|  | KT 2.5 | 1 | 4 | 5 |
|  | KT 2.6 | 4 | 4 | 8 |
|  | KT 2.7 | 2 | 3 | 5 |
|  | KT 2.8 |  | 3 | 3 |
|  | KT 2.9 | 1 | 4 | 5 |
|  | KT 3.0 | 4 | 2 | 6 |
|  | KT 3.1 | 4 | 4 | 8 |
|  | KT 3.2 | 4 | 4 | 8 |
|  | KT 3.3 | 3 | 4 | 7 |
|  | KT 3.4 | 3 | 4 | 7 |
|  | KT 3.5 | 4 | 4 | 8 |
|  | KT 3.6 | 4 | 4 | 8 |
| Kawanda | 238/29/1 | 4 | 4 | 8 |
|  | 267s/25/7 | 3 | 4 | 7 |
|  | KW 0.1 | 4 | 4 | 8 |
|  | KW 0.2 | 4 | 4 | 8 |
|  | KW 0.3 | 1 | 4 | 5 |
|  | KW 0.6 | 1 | 4 | 5 |
|  | KW 0.8 |  | 4 | 4 |
|  | KW 0.9 |  | 2 | 2 |
|  | KW 1.0 | 4 | 4 | 8 |
|  | KW 1.1 | 4 | 4 | 8 |
|  | KW 1.2 | 4 | 4 | 8 |
|  | KW 1.4 | 1 | 4 | 5 |
|  | KW 1.5 | 2 | 3 | 5 |
|  | KW 1.6 | 4 | 4 | 8 |
|  | KW 1.7 | 4 | 4 | 8 |
|  | KW 1.8 |  | 4 | 4 |
|  | KW 1.9 | 4 | 4 | 8 |
|  | KW 2.0 | 4 | 4 | 8 |
|  | KW 2.1 | 4 | 4 | 8 |
| Mabira | MB 1.4 | 4 | 3 | 7 |
|  | MB 2.3 | 3 | 4 | 7 |
|  | MB 2.4 | 2 | 4 | 6 |
|  | MB 2.5 | 2 | 4 | 6 |
|  | MB 3.1 | 4 | 4 | 8 |
|  | MB 3.2 |  | 4 | 4 |
|  | MB 3.3 | 4 | 4 | 8 |
|  | MB 3.4 | 3 | 4 | 7 |
|  | MB 3.5 | 3 | 3 | 6 |
|  | MB 4.1 | 3 | 4 | 7 |
|  | MB 4.3 | 4 | 4 | 8 |
|  | MB 4.4 |  | 3 | 3 |
|  | MB 4.5 | 4 | 4 | 8 |
|  | MB 5.1 | 4 | 3 | 7 |
|  | MB 5.2 | 4 | 4 | 8 |
| Malabigambo | ML 2.1 | 4 | 4 | 8 |
|  | ML 2.3 | 4 | 4 | 8 |
|  | ML 2.4 | 4 | 4 | 8 |
|  | ML 5.1 | 4 | 4 | 8 |
|  | ML 6.1 | 4 | 4 | 8 |
|  | ML 6.2 | 4 | 4 | 8 |
|  | ML 6.3 | 4 | 4 | 8 |
| Zoka | ZK 1.1 | 3 | 4 | 7 |
|  | ZK 1.2 |  | 4 | 4 |
|  | ZK 1.3 | 3 | 4 | 7 |
|  | ZK 1.4 | 4 | 4 | 8 |
|  | ZK 1.5 | 3 | 4 | 7 |
|  | ZK 2.1 | 4 | 4 | 8 |
|  | ZK 2.2 |  | 2 | 2 |
|  | ZK 2.3 | 3 | 4 | 7 |
|  | ZK 2.4 | 4 | 4 | 8 |
|  | ZK 2.5 | 2 | 4 | 6 |
|  | ZK 3.1 |  | 4 | 4 |
|  | ZK 3.2 | 1 | 4 | 5 |
|  | ZK 3.3 |  | 2 | 2 |
|  | ZK 3.4 | 4 | 4 | 8 |
|  | ZK 3.5 | 2 | 4 | 6 |
|  | ZK 4.1 | 4 | 4 | 8 |
|  | ZK 4.2 | 3 | 4 | 7 |
|  | ZK 4.3 | 4 | 4 | 8 |
|  | ZK 4.4 |  | 4 | 4 |
|  | ZK 4.5 | 4 | 4 | 8 |
|  | ZK 5.1 | 2 | 3 | 5 |
|  | ZK 5.2 | 3 | 4 | 7 |
|  | ZK 5.3 | 1 | 4 | 5 |
|  | ZK 5.4 | 3 | 4 | 7 |
|  | ZK 5.5 |  | 4 | 4 |
| **Grand Total** |  | **374** | **545** | **919** |


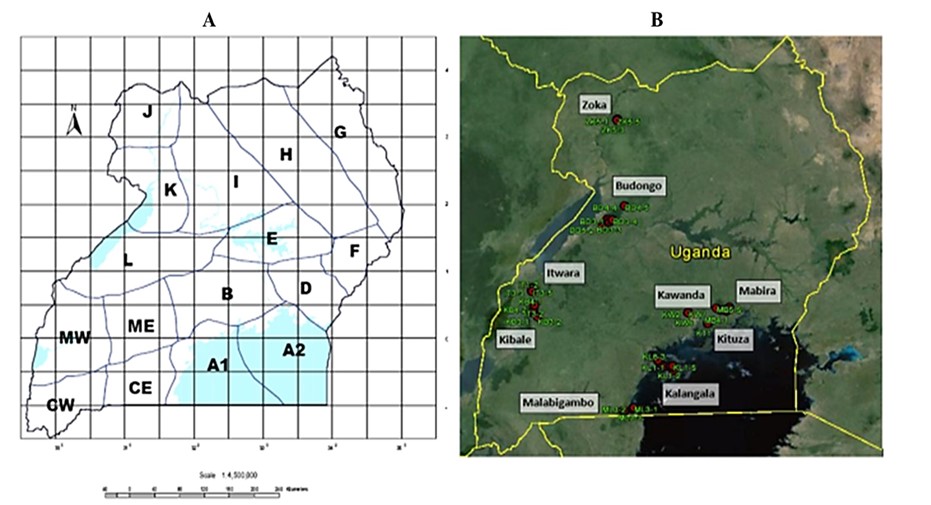


*Appendix Fig. A.1. Locations and the different climatic zones in which they occur; A: Location (climatic zone); Budongo (K), Itwara (L), Kalangala (A1), Kawanda (B), Kibale (L), Kituza (B), Mabira (B), Malabigambo (AI) and Zoka (J). B: Red and green indicate points of sample collection and codes of the samples respectively.*


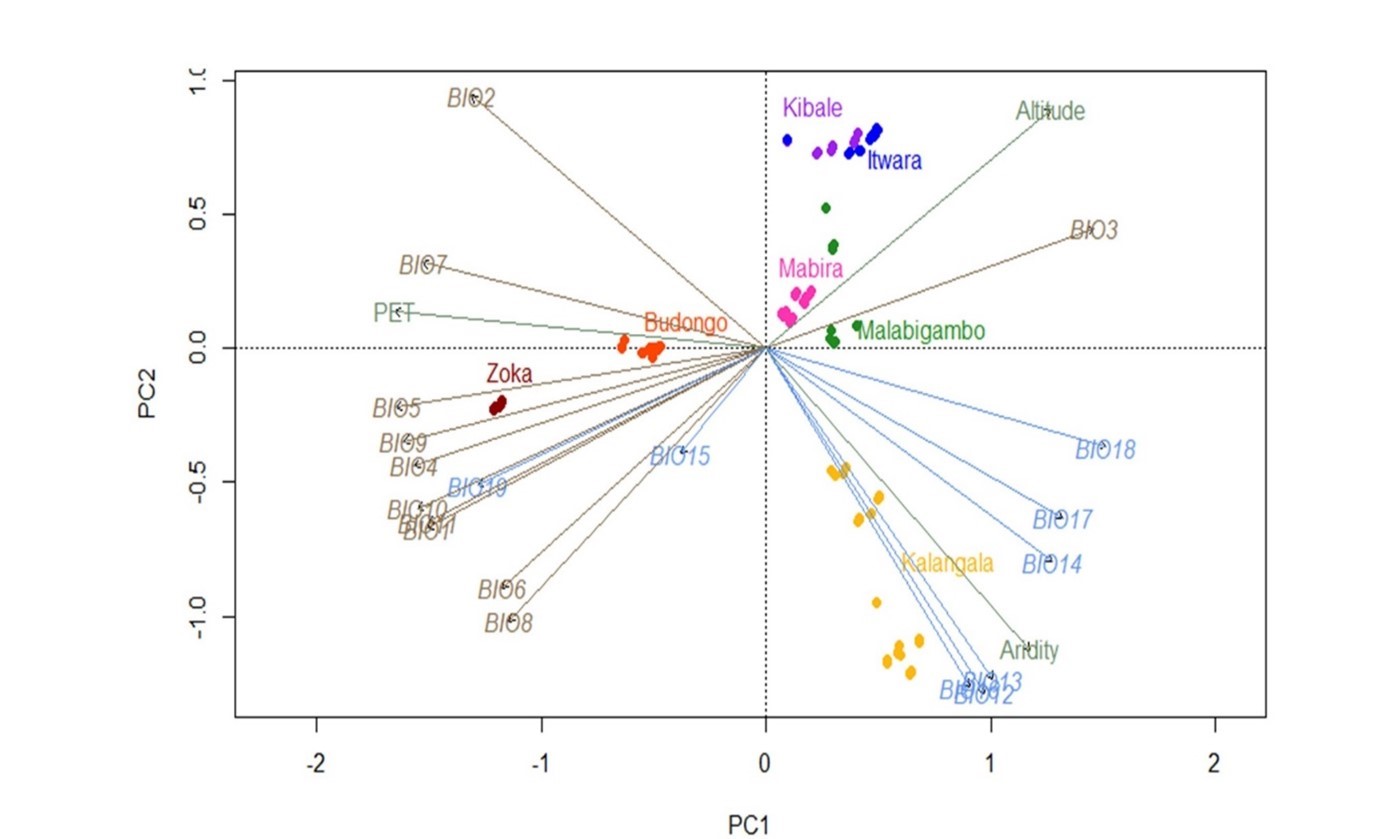


*Appendix Fig. A.2. Principal component analysis (PCA) of 19 bioclimatic variables: Temperature related variables coloured (cornflowerblue): Annual Mean Temperature (BIO1), Mean Diurnal Range (Mean of monthly (max temp - min temp)) (BIO2), Isothermality (BIO2/BIO7) (* 100) (BIO3), Temperature Seasonality (standard deviation *100) (BIO4), Max Temperature of Warmest Month (BIO5), Min Temperature of Coldest Month (BIO6), Mean Temperature of Wettest Quarter (BIO8), Mean Temperature of Driest Quarter (BIO9), Mean Temperature of Warmest Quarter (BIO10), Mean Temperature of Coldest Quarter (BIO11), precipitation related coloured (burlywood4): Annual Precipitation (BIO12), Precipitation of Wettest Month (BIO13), Precipitation of Driest Month (BIO14), Precipitation Seasonality (Coefficient of Variation) (BIO15), Precipitation of Wettest Quarter (BIO16), Precipitation of Driest Quarter (BIO17), Precipitation of Warmest Quarter (BIO18), Precipitation of Coldest Quarter (BIO19) and 3 other environmental variables, Altitude, Aridity (Wetness Index (WI)) and Potential Evapotranspiration (PET) coloured (darkseagreen4) at Ugandan C. canephora wild sites.*


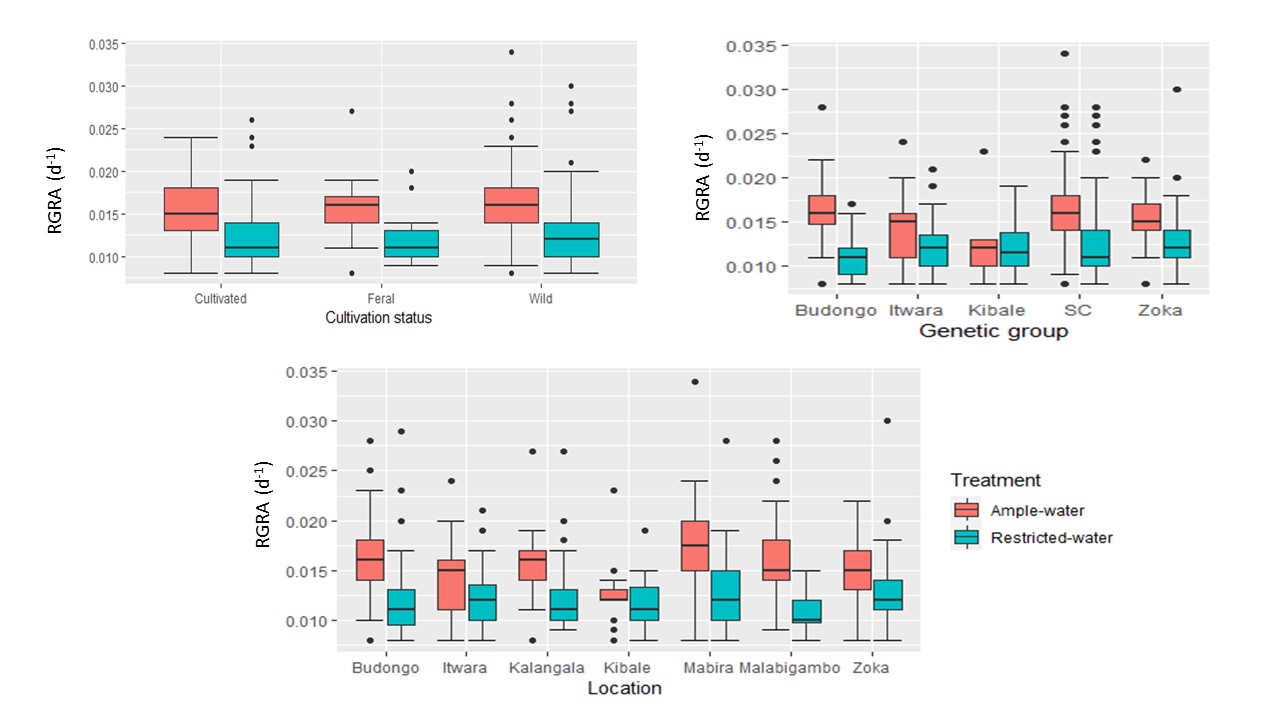
*Appendix Fig. A.3. Effect of water treatment on Relative growth rate in leaf area (RGRA[d^-1^]) across cultivation status (A), genetic groups (B) and location (C)*


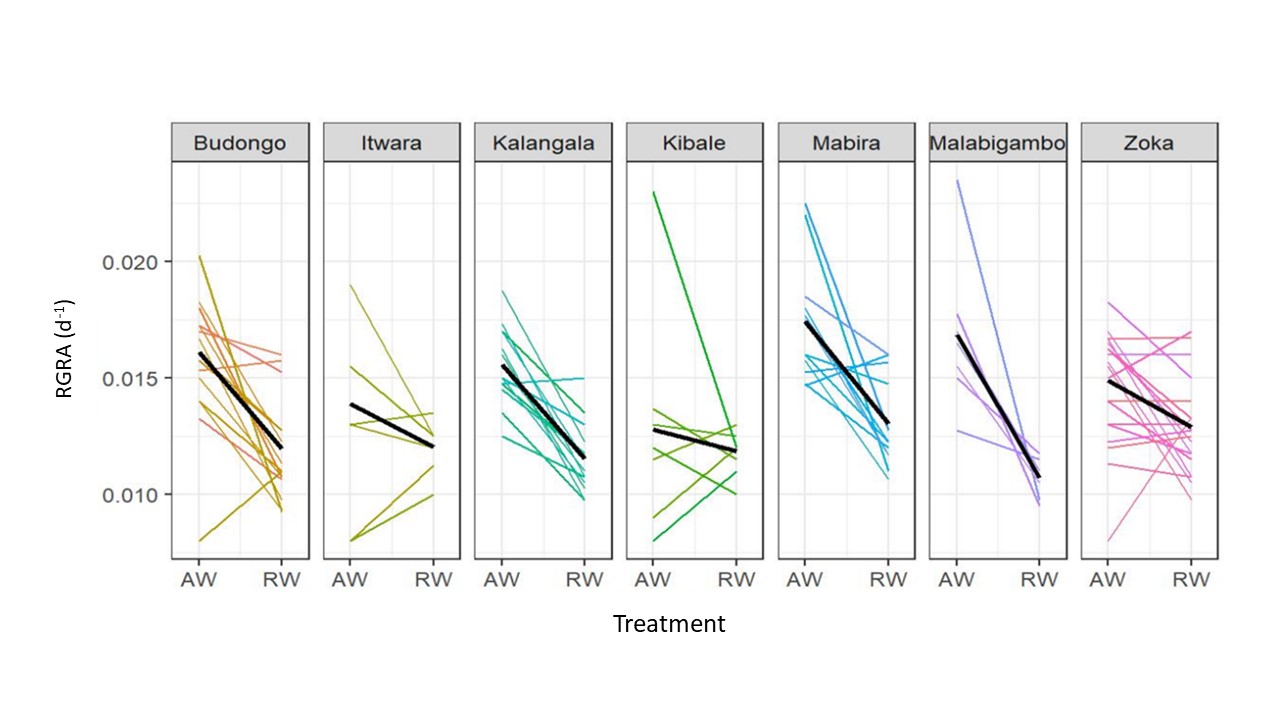


*Appendix Fig. A.4. Mean RGRA [d^-1^] as a function of treatment (ample-water (AW) and restricted-water (RW) across location (panels) and genotypes (coloured lines). Solid black line shows the mean estimated response per location.*


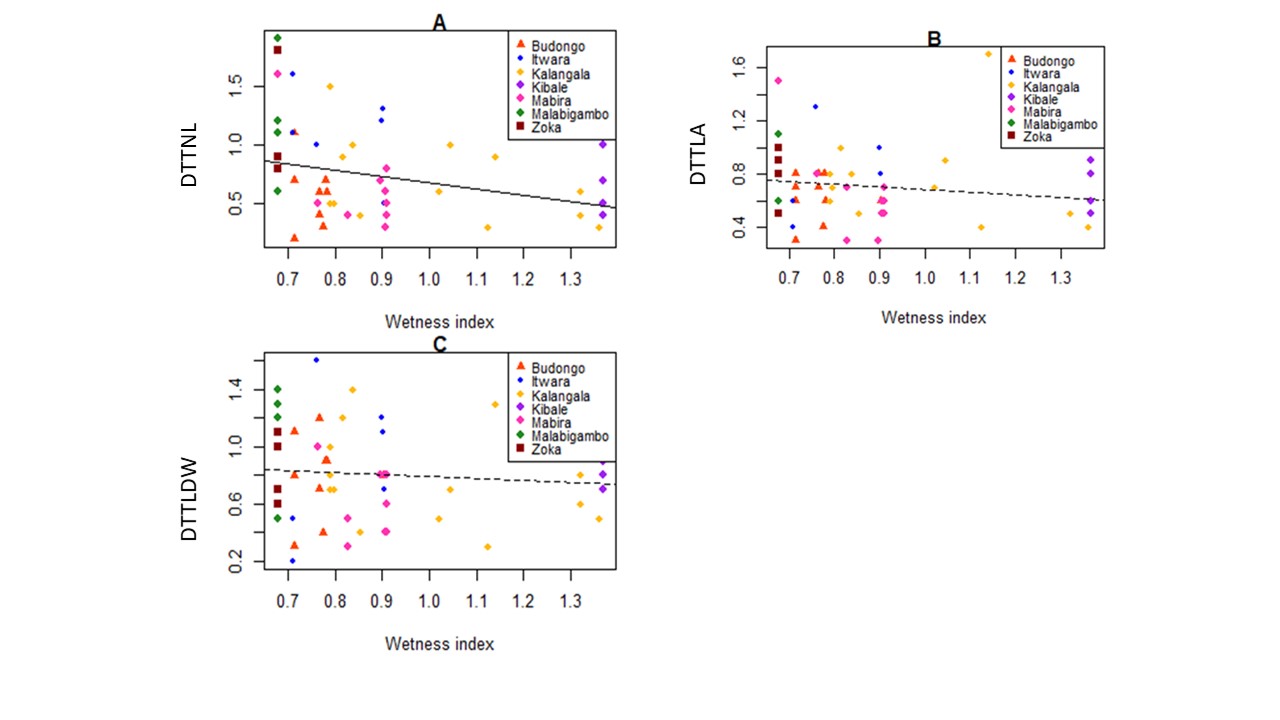


Appendix Fig. A.5. Relationship between tolerance of growth traits in *C. canephora* genotypes and wetness index of the location in which they were collected from (A) Drought tolerance in Total Number of leaves (DTTNL) and wetness index (B); Drought tolerance in Total Leaf area (DTTL) and wetness index (C) Drought tolerance in Total Leaf Dry Weight (DTTLDW) and wetness index, high wetness index values indicate moist conditions and low WI values indicate dry conditions


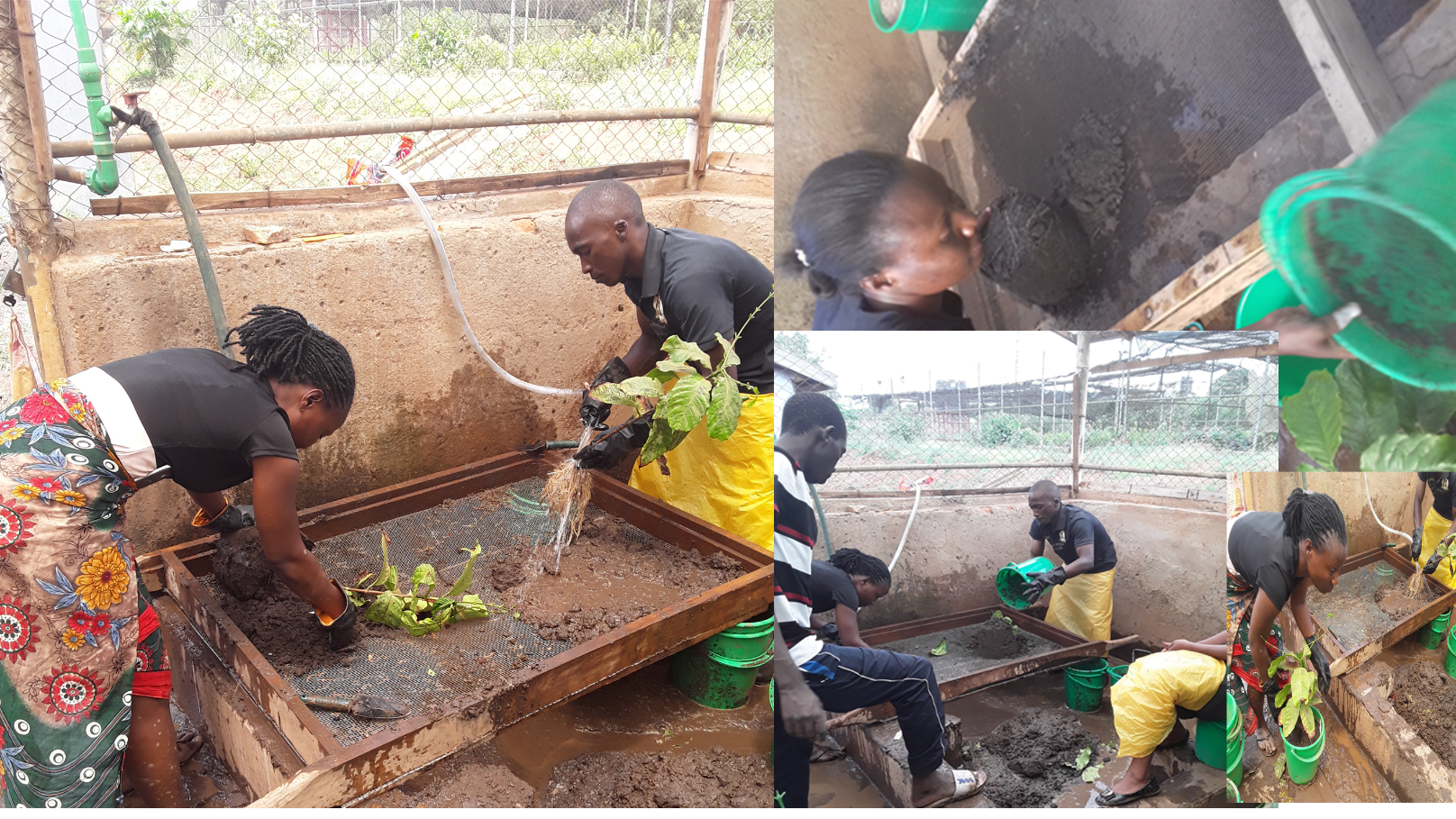


Appendix Plate A1. Photos showing the root extraction process and evidence of minimal or no pot-binding effect.


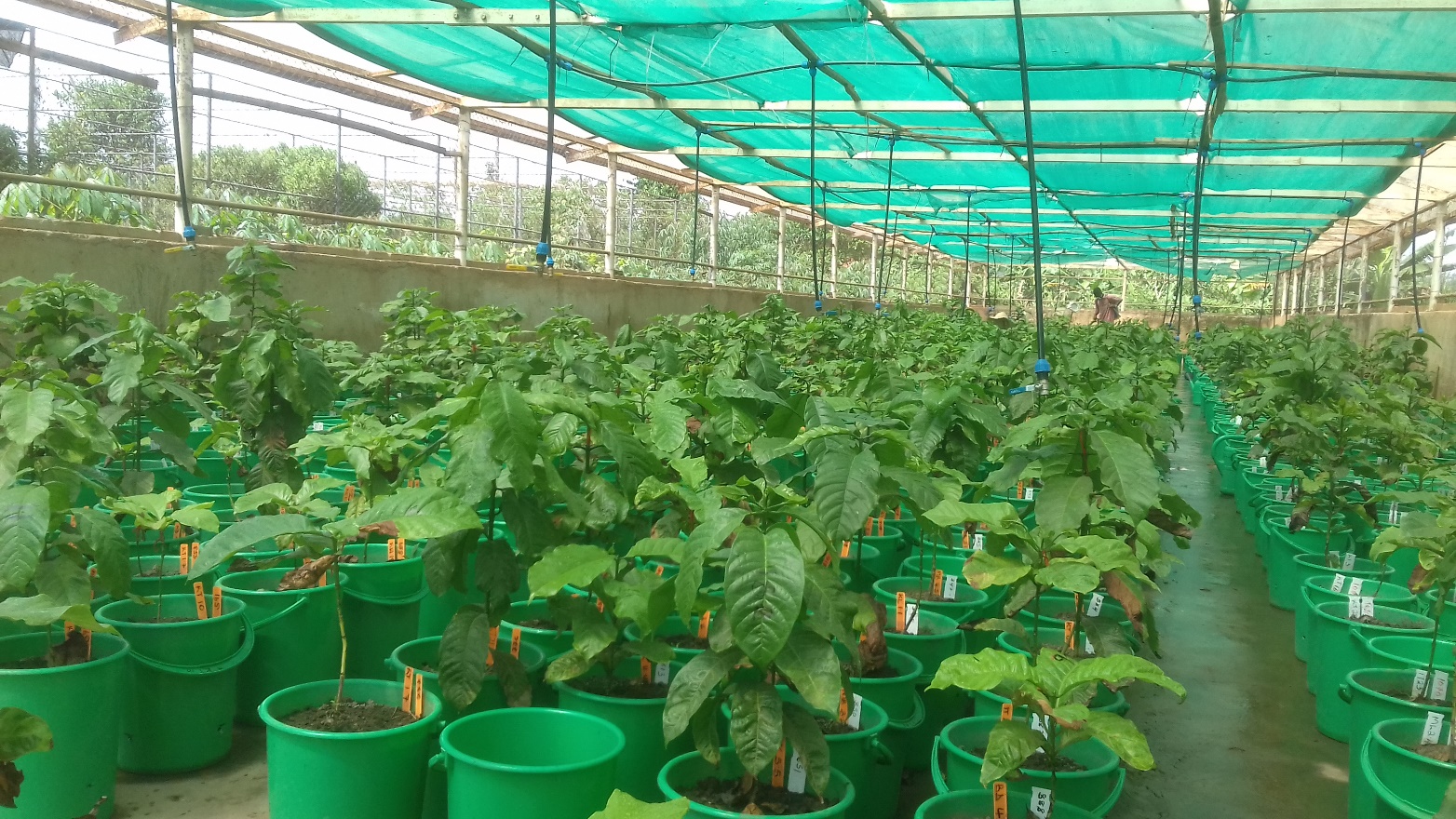


Appendix Plate A2. Experimental shelter overview and size of the plants at final harvest.
